# Ablation versus medical therapy for patients with atrial fibrillation: An updated meta‐analysis

**DOI:** 10.1002/clc.24184

**Published:** 2023-11-08

**Authors:** Fatemeh Kheshti, Saeed Abdollahifard, Alireza Hosseinpour, Mehdi Bazrafshan, Armin Attar

**Affiliations:** ^1^ Department of Cardiovascular Medicine, School of Medicine Shiraz University of Medical Sciences Shiraz Iran; ^2^ Students' Research Committee Shiraz University of Medical Sciences Shiraz Iran; ^3^ Research Center for Neuromodulation and Pain Shiraz Iran

**Keywords:** antiarrhythmic drugs, atrial fibrillation, catheter ablation, medical therapy

## Abstract

To investigate the effect of ablation compared to medical therapy on clinical outcomes of patients with atrial fibrillation (AF). PubMed, Scopus, Embase, and Web of Science databases were searched using ablation, medical treatment, AF, and related words. The effect of ablation and medical therapy was sought to be gathered on stroke or transitional ischemic attack, mortality, hospitalization, recurrence of AF, progression of AF, and left ventricular ejection fraction. Analyses were performed using R software. 31 studies (the results of 27 randomized controlled trials), compromising an overall 6965 patients (Ablation, *n* = 3643; Medical treatment, *n* = 3322) were reviewed in our study, revealed that catheter ablation would result in substantial benefits for patients with AF without significant difference in serious adverse events compared to medical management (Risk Ratio: 0.92, [95% Confidence Interval (CI), 0.64−1.33]). Catheter ablation in patients with AF significantly resulted in a 29% reduction in all‐cause mortality (RR: 0.71, [95% CI, 0.57−0.88]), a 57% reduction in hospitalization (RR: 0.43, [95% CI, 0.27−0.67]), a 53% reduction in AF recurrence (RR: 0.47, [95% CI, 0.36−0.61]), and a dramatic reduction, 89%, in progression of paroxysmal to persistent AF (RR: 0.11, [95% CI, 0.02−0.65]); also associated with a remarkable improvement in their left ventricular ejection fraction (LVEF) (Mean Difference, MD: 6.84%, [95% CI, 3.27−10.42]) compared to medical therapy. Our study showed that ablation may be superior to medical therapy in patients with AF regarding AF recurrence, mortality, LVEF improvement, hospitalization, and AF progression outcomes.

## Graphical abstract

Key question

Ablation or medical management for atrial fibrillation (AF)?

Key findings

No significant difference in serious adverse events rate.

Lower risk of atrial arrythmia recurrence, progression to persistent AF, and hospitalization.

Better left ventricular ejection fraction improvement with ablation in heart failure with reduced ejection fraction patients.

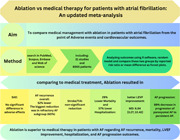

**Abbreviations**
A: ablation
AA: atrial arrhythmia
AAD: antiarrhythmic drugs
AF: atrial fibrillation
AFL: atrial flutter
AT: atrial tachyarrhythmia
BMI: body mass index
BMT: best medical treatment
CA: catheter ablation
CI: confidence interval
CRT‐D: cardiac resynchronization therapy defibrillator
CV: cardiovascular
CVH: cardiovascular hospitalization
DM: diabetes mellitus
ECG: electrocardiogram
EF: ejection fraction
HF: heart failure
HFH: heart failure hospitalization
HFrEF: heart failure with reduced ejection fraction
HR: hazard ratio
ICD: implantable cardioverter defibrillator
IQR: interquartile range
IRR: incidence rate ratio
LA: left atrium
LAD: left atrial diameter
LS PAF: long standing persistent atrial fibrillation
LV: left ventricle
LVEF: left ventricular ejection fraction
MD: mean difference
MRC: medical rate control
NYHA: New York Heart Association functional class of heart failure
N.: number of
PAF: paroxysmal atrial fibrillation
PVI: pulmonary vein isolation
PWI: posterior wall isolation
RCT: randomized controlled clinical trial
RF: radiofrequency
RF CA: radiofrequency catheter ablation
RR: risk ratio
SAE: serious adverse events
SD: standard deviation
TIA: transient ischemic attack
TTM: Trans‐Telephonic Monitor

## 1 INTRODUCTION

Atrial fibrillation (AF) is a supraventricular tachyarrhythmia caused by chaotic atrial activation resulting in ineffective atrial contraction which is known to be the most prevalent sustained cardiac arrhythmia in adults.^1^ Medical problems caused by or related to AF can increase morbidity and mortality rate affecting patients' quality of life and life expectancy. Heart failure (HF), stroke, peripheral thromboembolism, cardiomyopathy, renal failure, myocardial infarction, dementia, and death are seen to be related to AF.^1^, ^2^ For such problems caused by AF, management of this disease is one of the medical concerns. Rate control achievement with betablockers and calcium channel blockers can help reduce patients' symptoms and prevent adverse cardiovascular outcomes such as HF and cardiomyopathy induced by tachycardia.^3^, ^4^ Rhythm control plays a significant role in AF outcomes as it was seen that AF progression was significantly lower in patients with obtained sinus rhythm. As, the effectiveness of drug therapy ranges between 39% and 63% for sinus rhythm achievement and considering the adverse drug effects resulting in discontinuation of therapy (30%), using ablation as initial therapy has obtained attention.^1^, ^5^, ^6^, ^7^, ^8^, ^9^ In a recent randomized controlled clinical trial (RCT) conducted to find if there is a superiority of cryo‐balloon ablation over antiarrhythmic drugs (AADs) as initial therapy for symptomatic paroxysmal AF, was seen that AF recurrence was significantly lower.^5^, ^8^, ^10^ However, in another RCT which used radiofrequency ablation instead of cryo‐balloon, there was no significant difference between the treated groups who received ablation and AADs as initial therapy.^11^ Consequently, the best treatment is still controversial.

Some meta‐analyses have been done to compare outcome of patients with AF, whether treated by catheter ablation or medical therapy, and the results showed some inconstancy. Evaluation of previous meta‐analysis showed that most recent and very large trials^10^, ^12^, ^13^ have not been included in some of them.^14^, ^15^ In another recent meta‐analysis, progression of AF was not evaluated between medical treatment and ablation, or the analyzed data were inconsistent.^16^ Furthermore, the results from some others were contradictory. For example, two different previous meta‐analysis done by Mao et al. and Asad et al. resulted in nonsignificant stroke/TIA risk reduction in CA group.^14^, ^15^ On the contrary, Song et al. in their new meta‐analysis resulted that the effect of CA on risk of stroke/TIA in AF patients could assumed to be significant (RR: 0.61, [0.39−0.96]).^16^ Considering these controversies and inconsistencies, performing a new meta‐analysis that covers all aspects of ablation vs medical treatment for treatment of patients with AF and includes all new studies in this field, evaluates all the subgroups and outcomes simultaneously in one study seemed to be essential. Here, we aimed to perform a comprehensive meta‐analysis not only to address these conflicts but also to update the current evidence and analysis of subgroups of patients to have a better insight into the effects of ablation and medical therapy for patients with AF especially by considering various subgroups including cryo‐balloon or radiofrequency ablation techniques. Also in this comprehensive meta‐analysis, for the first time Progression of AF was evaluated as one of the secondary end points of comparing these two different managements.

## 2 METHODS

This study has been conducted according to Preferred Reporting Items for Systematic Reviews (PRISMA) 2020.

### 2.1 Eligibility criteria

RCTs that compared catheter ablation (radiofrequency or cryosurgery) with medical treatment for patients with AF were considered eligible for review. Exclusion criteria were case series, editorials, erratum, letters to the editor, narrative reviews, conference abstracts, and retracted articles.

### 2.2 Information sources and search strategy

PubMed, Scopus, Embase, and Web of Science databases were searched on December 24th of 2021 using a combination of the words “Radiofrequency Ablation,” Cryosurgery,” “Catheter Ablation,” “Atrial Fibrillation,” “Best medical treatment,” and their related synonyms. The title/abstract filter was the only filter that was used, and no automated search tool was used for the search process. The comprehensive search strategy could be found in the Supporting Information Material.

### 2.3 Selection process and data collection process

The yield of our search was exported into an endnote library (Version X 8, Clarivate Analytics) and then duplicates were omitted manually. The screening process was started using the Rayyan web‐based tool.^17^ For screening two separate authors (A. S. and F. K.) were involved and conflicts were instigated by a third author (A. A.) at that application. In this step of screening, titles and abstracts were investigated for meeting our inclusion and exclusion criteria. In the second step, included articles were exported from the Rayyan app into an endnote library and full texts were added and screened.

### 2.4 Data extraction

An excel spread sheet has been created to extract the outcomes of the eligible articles. The desirable outcomes were stroke or transitional ischemic attack, mortality, hospitalization, recurrence of AF or any atrial tachyarrhythmia (AT), progression of AF all as events, and change in the mean of left ventricular ejection fraction (LVEF). The data extraction was first performed by one author and then rechecked by two other researchers. The effect of the intervention in patients with HF, paroxysmal, persistent, naive, or refractory AF and different types of catheter ablation like cryo‐balloon and radiofrequency were subsets that were sought to be gathered as subgroups.

### 2.5 Risk of bias assessment

The risk of bias for RCTs was assessed using RevMan (version 5.4, Cochrane)^18^ and reported as two separate figures. This tool classified each domain into high risk, low risk, and unclear risk of bias.

### 2.6 Synthesis methods

The core analysis was carried out using R software (Version 4).^19^ A Random‐effect model was considered for analysis. Heterogeneity was assessed using *I* ^2^, and heterogeneity of more than 50% was considered significant. Pooled data and subgroups were reported in a forest plot. Publication bias was assessed using funnel plots and quantitively using Egger's test (for continuous data) and Petter's test (for binary data) for analyses that included nine and more studies. A *p* Value of less than .05 in both tests was considered as a significant publication bias and a trim and fill test was performed to consolidate the results in cases that any significant publication bias was detected. Sensitivity analysis was performed using the leave‐one‐out method.

## 3 RESULTS

### 3.1 Study selection

**Figure 1:**
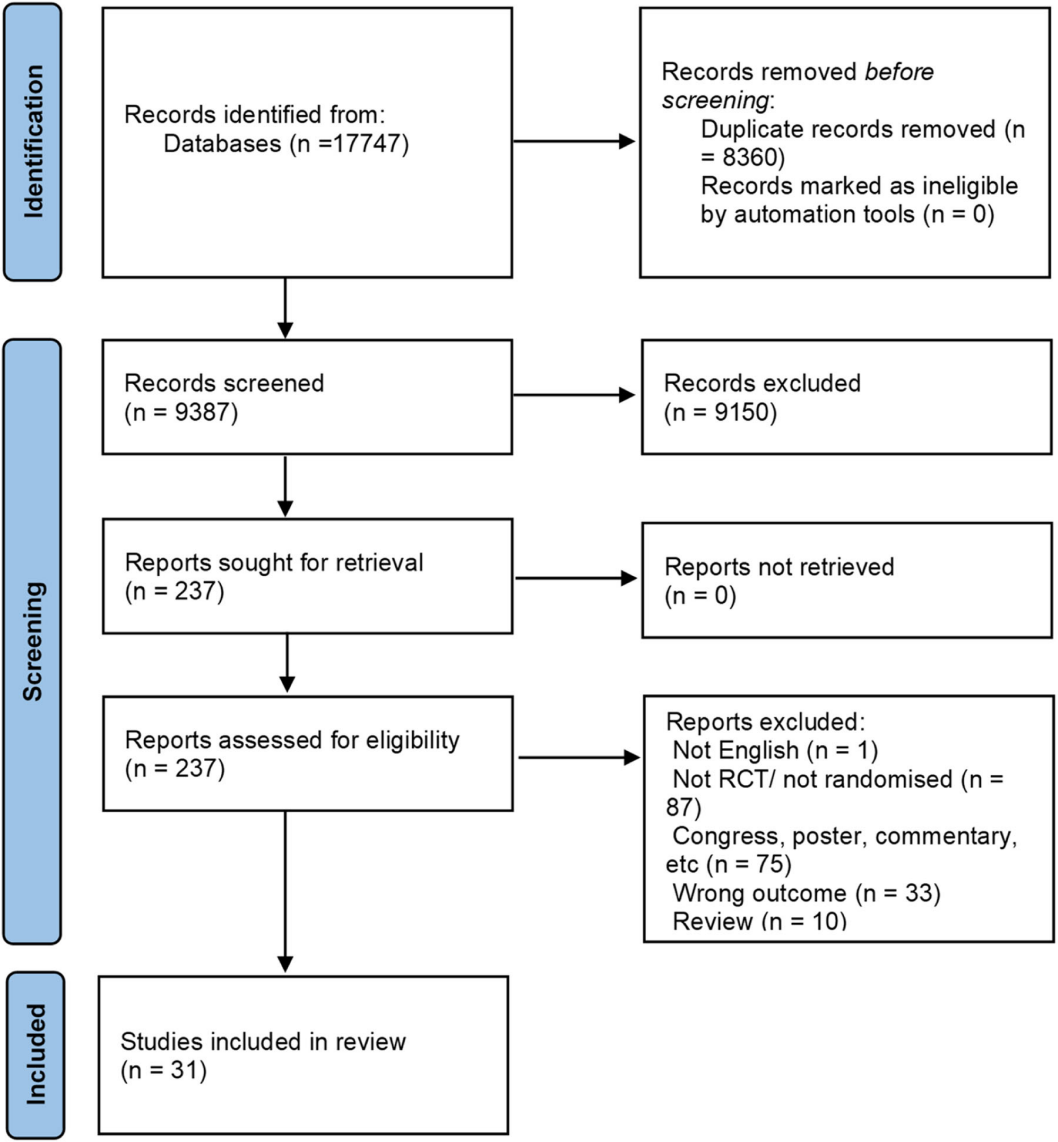
PRISMA flowchart included studies investigating the effect of ablation compared to medical treatment in management of patients with atrial fibrillation.

Our search emerged in 17 747 articles and after removing duplications, 9387 articles remained for the two‐step screening process. The screening process ensued in 31 articles selected for our current study for further evaluation. Finally, 31 articles (from 27 different RCTs) were incorporated in this systematic review. PRISMA flowchart clarifies the comprehensive study selection process (Figure 1).

### 3.2 Study characteristics

All selected studies for this meta‐analysis were RCTs. 31 articles were included in our study which were the results of 27 different RCTs. We have reviewed a total number of 6965 patients (ablation: 3643, best medical treatment, BMT: 3322), including 1330 patients with HF with reduced ejection fraction (HFrEF; ablation: 655, BMT; 675), 1220 AF patients who did not receive any AADs before the trial and therefore were naïve‐treatment for AF (ablation: 615, BMT; 605), 1653 patients who had failed to at least one AAD before study and therefore assumed as refractory AF (ablation: 962, BMT; 691). There were 12 studies almost assessing just patients with paroxysmal AF,^5^, ^8^, ^9^, ^10^, ^11^, ^20^, ^21^, ^22^, ^23^, ^24^, ^25^, ^26^ 10 studies just studying patients with persistent or long‐standing persistent AF,^27^, ^28^, ^29^, ^30^, ^31^, ^32^, ^33^, ^34^, ^35^, ^36^ and the remainder were a mixture of three different AF types.

The ablation method in the majority of our selected studies was radiofrequency catheter ablation, except for one study using hot balloon ablation,^26^ and four other studies using cryo‐balloon ablation.^5^, ^8^, ^10^, ^37^ The comparator medical therapy group in most of the selected studies used AADs, while four trials used the rate control medications,^29^, ^30^, ^32^, ^35^ and three trials used a combination of both.^7^, ^31^, ^38^

### 3.3 Risk of bias in studies

Quality and risk of bias assessment have been illustrated in Supporting Information S1: Figure 1 in the Supporting Information Material.

#### 3.3.1 Serious adverse events (SAE)

**Figure 2:**
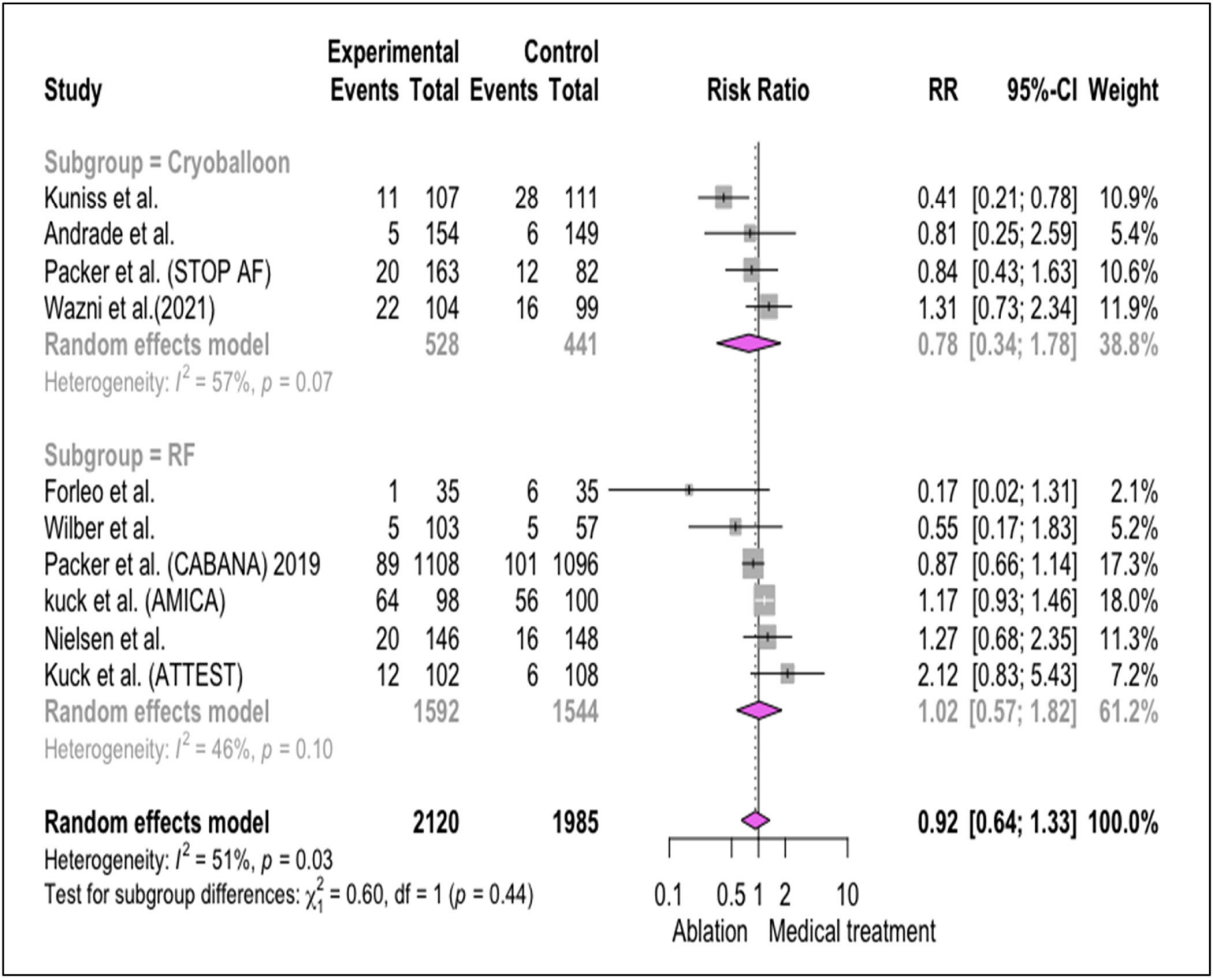
Forest plot of studies comparing medical therapy with two different methods of ablation in term of SAE in AF patients (radio‐frequency catheter ablation vs. BMT/cryo‐balloon catheter ablation vs. BMT). AF, atrial fibrillation; SAE, serious adverse events.

Analysis of 10 studies has been performed for comparing risk ratio (RR) of SAE in the ablation group versus BMT, revealed that there was not any significant differences between these two methods of treatment (RR: 0.92, [0.64−1.33], *I* ^2^: 51%, *p* = .03).^5^, ^8^, ^10^, ^11^, ^21^, ^31^, ^37^, ^38^, ^39^, ^40^ For comparing cryo‐balloon and radiofrequency catheter ablation (RF CA) as two different methods of ablation, subgroup analysis was done and showed lower SAE in trials that used cryo‐balloon ablation (0.78; [0.34−1.78], *I* ^2^: 57%, *p* = .07) compared to RF CA (1.02, [0.57−1.82], *I* ^2^: 46%, *p* = .1).^5^, ^8^, ^10^, ^37^, ^11^, ^21^, ^31^, ^38^, ^39^, ^40^ (*Forest plots of SAE outcome have been illustrated in* Figure 2).

#### 3.3.2 Atrial arrhythmia recurrence

**Figure 3:**
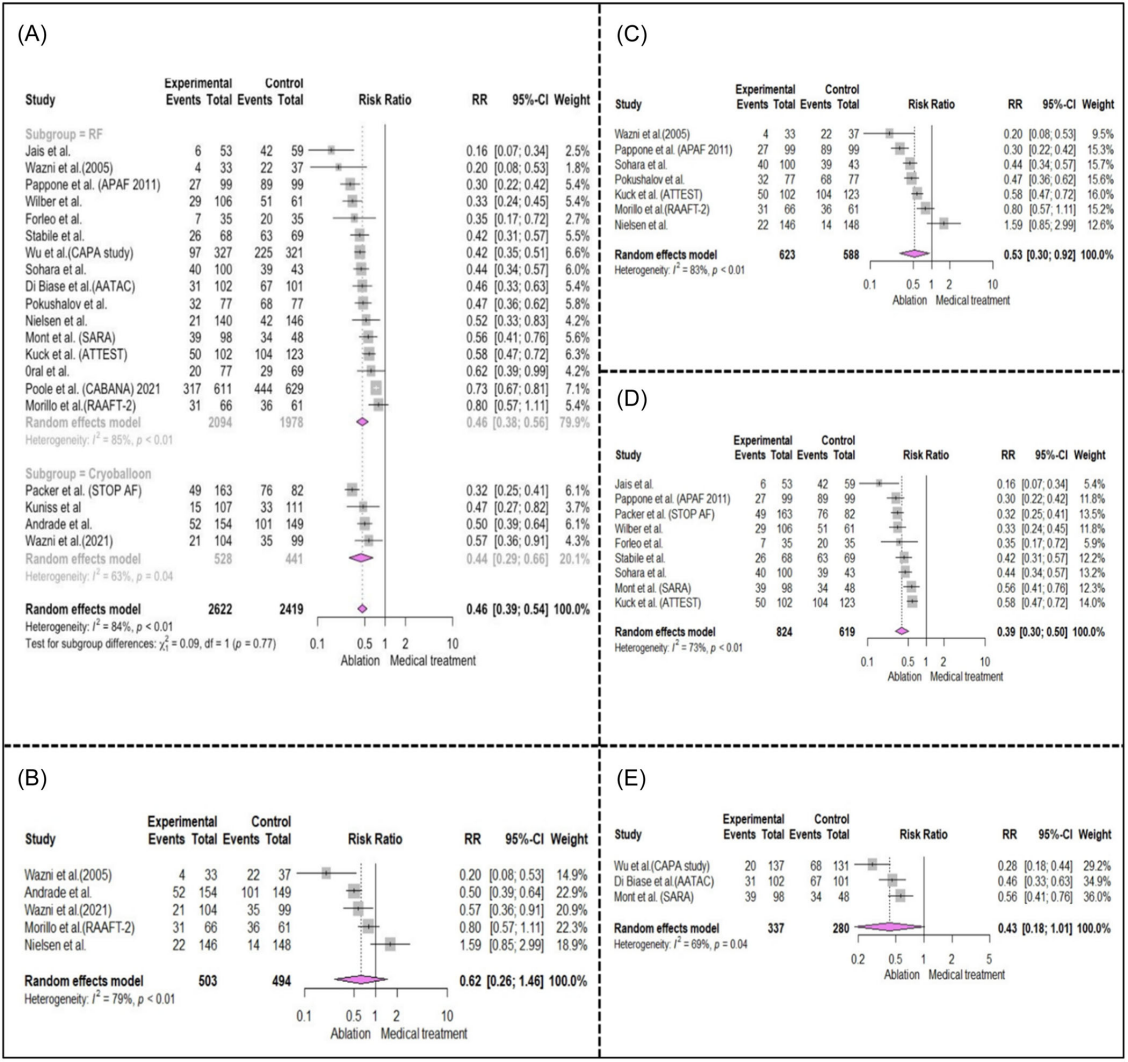
Forest plot of included studies investigating the effect of ablation on atrial arrhythmia recurrence compared to medical treatment in several subgroups. (A: Comparing common methods of ablation, B−E: subgroup analysis in different atrial fibrillation [AF] subtype). (A) Cryo‐balloon and radiofrequency catheter ablation. (B) Naïve AF (did not receive any treatment for their AF condition before the trial). (C) Paroxysmal AF. (D) Refractory AF (patients with AF who had failed at least one AAD before trial). (E) Persistent AF.

Analysis of 20 studies comparing the effect of ablation versus BMT on the recurrence of any atrial arrythmia (AF, AT, AFL),^5^, ^8^, ^9^, ^10^, ^11^, ^13^, ^20^, ^21^, ^22^, ^24^, ^25^, ^26^, ^27^, ^33^, ^36^, ^37^, ^39^, ^40^, ^41^, ^42^ and 12 studies on the recurrence of AF,^8^, ^9^, ^11^, ^13^, ^20^, ^22^, ^26^, ^27^, ^33^, ^36^, ^39^, ^42^ showed that ablation was dramatically more effective (RR: 0.46, [0.39−0.54], *I* ^2^: 84%, *p* < .01 and RR: 0.47, [0.36−0.61], *I* ^2^: 83%, *p* < .01, respectively), especially in refractory AF (RR: 0.39, [0.30−0.50], *I* ^2^: 73%, *p* < .01 for AA recurrence and RR: 0.40, [0.24−0.70], *I* ^2^: 59%, *p* = .05 for AF recurrence),^20^, ^21^, ^24^, ^26^, ^33^, ^37^, ^39^, ^40^, ^42^ and persistent AF subgroup (RR: 0.46, [0.32−0.66], *I* ^2^: 19%, *p* = .29 for AF recurrence) ^27^, ^33^, ^36^ (Figure 3). Ablation could also be preferred as the treatment of choice in naïve‐treatment PAF patients as ablation was significantly more effective on preventing the recurrence of atrial arrhythmia compared to BMT (RR: 0.53, [0.37−0.76], *I* ^2^: 50%, *p* = .080).

Finally, our subgroup analyses for comparing cryo‐balloon ablation with RF CA did not result in a significant difference in AA recurrence between these two methods (cryo‐balloon ablation vs. BMT, RR: 0.44, [0.29−0.66], *I* ^2^: 63%, *p* = .04) (RF CA vs. BMT, RR: 0.46, [0.38−0.56], *I* ^2^: 85%, *p* < .01).^5^, ^8^, ^10^, ^37^, ^9^, ^11^, ^13^, ^20^, ^21^, ^22^, ^24^, ^25^, ^26^, ^27^, ^33^, ^36^, ^39^, ^40^, ^41^, ^42^

**Figure 4:**
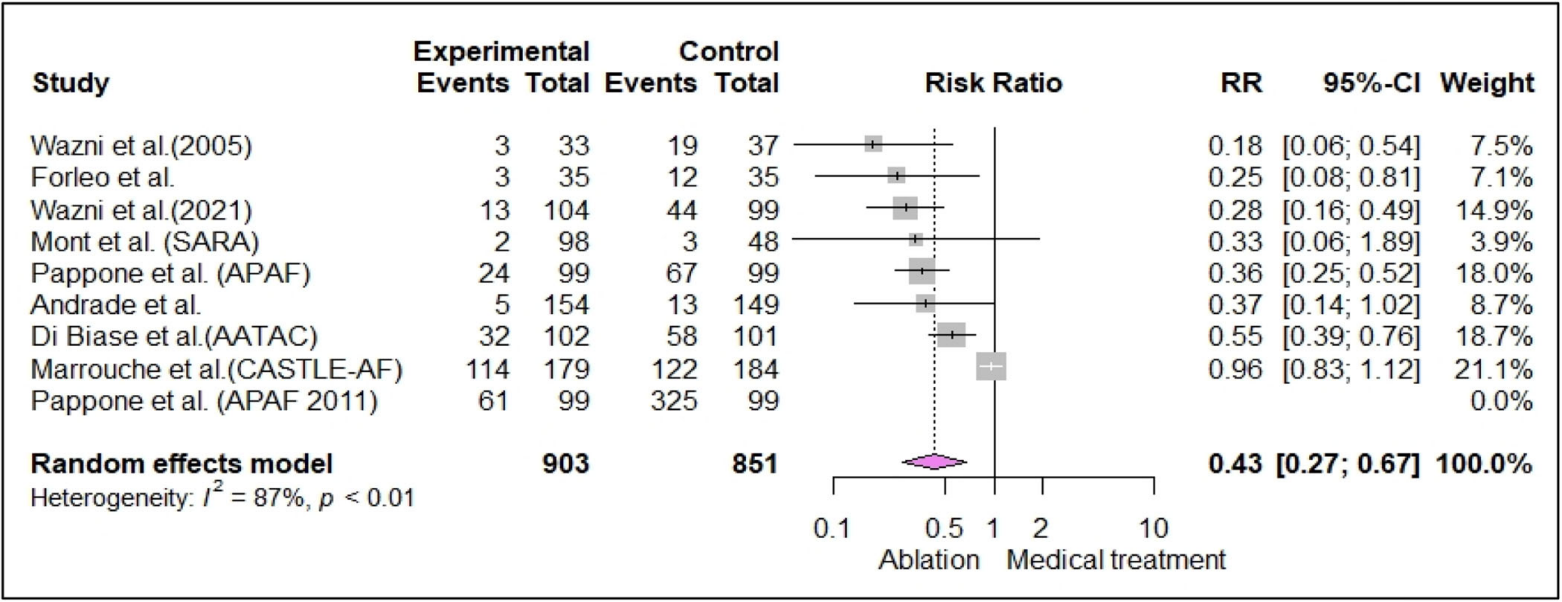
Forest plot of included studies investigating the effect of ablation on hospitalization compared to medical treatment.

**Figure 5:**
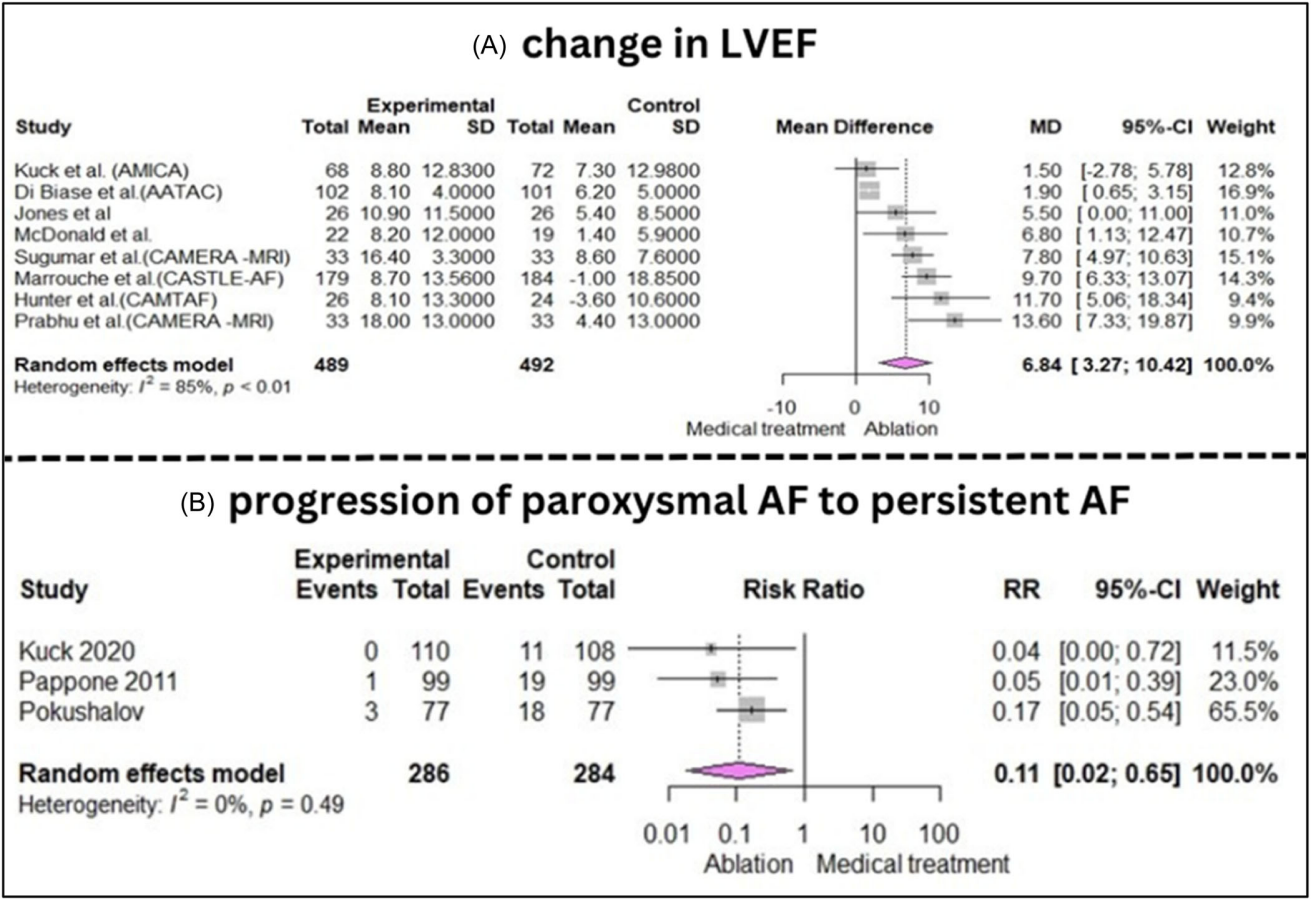
(A) Forest plot of studies comparing Ablation versus medical therapy in term of LVEF improvement in AF patients. (B) Forest plot of studies comparing Ablation versus medical therapy in term of progression of paroxysmal AF to persistent AF. AF, atrial fibrillation; LVEF, left ventricular ejection fraction.

*(Forest plots of AF recurrence outcomes have been illustrated in Supporting Information S1: Figures* 2–5 *in the Supporting Information Material)*.

#### 3.3.3 Stroke/TIA

Analysis of 12 studies for determining the overall effect of the two interventions on stroke and/or TIA revealed a nonsignificant reduction (RR: 0.71 [95% CI, 0.44−1.14], *I* ^2^: 0%, *p* = .57). The interventions' effect on stroke/TIA only in studies with a minimum follow‐up of 12 months revealed the same result (RR: 0.62, [0.37−1.04], *I* ^2^: 0%, *p* = .57).^5^, ^7^, ^11^, ^21^, ^36^, ^37^, ^38^, ^42^

*(Forest plots of Stroke/TIA outcomes have been illustrated in Supporting Information S1: Figures* 6−9 *in the Supporting Information Material)*.

#### 3.3.4 Mortality and hospitalization

Analysis of 11 studies showed that ablation could significantly reduce the mortality rate of AF patients compared to BMT (RR: 0.71, [0.57−0.88], *I* ^2^: 0%, *p* = .78).^7^, ^11^, ^20^, ^27^, ^28^, ^29^, ^31^, ^36^, ^37^, ^38^, ^42^ *(Forest plots of mortality outcome have been illustrated in Supporting Information S1: Figures* 10−13 *in the Supporting Information Material)*.

Analyses showed that ablation could be considered the superior therapy for reducing the hospitalization events in AF patients (RR: 0.43, [0.27−0.67], *I* ^2^: 87%, *p* < .01).^5^, ^7^, ^8^, ^9^, ^23^, ^24^, ^27^, ^33^, ^39^ The superlative effect was in AF patients who were receiving ablation as their first‐line management of AF by RR reduction of 72% (patients who had not received any AAD therapy before trials, Figure 4) (RR: 0.28, [0.14−0.54], *I* ^2^: 0%, *p* = .62).^5^, ^8^, ^9^ Ablation was superior to medical therapy in paroxysmal AF patients (RR: 0.32, [0.23−0.46], *I* ^2^: 0%, *p* = .63),^5^, ^8^, ^9^, ^23^, ^24^ and also in AF patients who were refractory to at least one AAD (RR: 0.35, [0.25−0.47], *I* ^2^: 0%, *p* = .85),^23^, ^24^, ^33^, ^39^ from the point of reducing hospitalization events.

*(Forest plots of the hospitalization outcome have been illustrated in Supporting Information S1: Figures* 14−17 *in the Supporting Information Material)*.

#### 3.3.5 Change in left ventricular ejection fraction

Analysis of different studies showed that comparing Ablation versus BMT in the management of AF showed that catheter ablation also resulted in a remarkable greater improvement in LVEF in a follow‐up range of 6 to 60 months (mean difference, MD: 6.84, [95% CI; 3.27−10.42], *I* ^2^: 85%, *p* < .01).^7^, ^27^, ^29^, ^30^, ^31^, ^32^, ^34^, ^35^ Improving LVEF especially was beneficial for persistent AF patients with concordant HFrEF (MD: 6.39, [2.26−10.53], *I* ^2^: 82%, *p* < .01).

*(Forest plots of the LVEF improvement outcome have been illustrated in Figure* 5A, *and Supporting Information S1: Figure* 18 *in the Supporting Information Material)*.

#### 3.3.6 AF progression

Analysis of 3 RCTs revealed that ablation is the superior management for paroxysmal AF compared to medical therapy, as it significantly delays the progression of the condition to persistent AF with a RR reduction of 89% (RR: 0.11, [0.02−0.65], *I* ^2^: 0%, *p* = .49)^21^, ^24^, ^25^ (Figure 5B).

### 3.4 Publication bias

Publication bias was only significant for the analysis of the investigation of the effect of ablation on AF recurrence events compared to medical treatment. A Trim and fill test was carried out and resulted in an adjusted RR of 0.652 with a 95% CI of 0.455−0.934.

## 4 DISCUSSION

To the best of our knowledge, this is the largest meta‐analysis that compared the effect of ablation with medical treatment in patients with AF and is the only one addressing the effect of cryo‐balloon ablation in the subgroup analyses. Analyses revealed that AF ablation results in a significantly lower mortality rate, hospitalization, AF recurrence, or any atrial arrhythmia recurrence and a further improvement in LVEF compared to medical management. These effects were both valid using cryo‐balloon and radiofrequency ablation methods while the risk of serious side effects is not increased significantly. Data on cardiovascular hospitalization and HF hospitalization was not adequate for meta‐analysis.

Our study showed that ablation would result in a significantly greater reduction in AF or any AA recurrence in patients with refractory, paroxysmal, or persistent AF. It is probable that this decrement in atrial arrhythmia recurrence would result in better patients' quality of life and exercise capacity tolerance, lower morbidity and complications. This study revealed that ablation would cause a remarkable 47 percent risk reduction of atrial arrhythmia recurrence in patients with PAF who were naïve to treatment.

The significant beneficial effect of ablation on recurrence of AA in the naïve subgroup in our study was in agreement with Andrade et al. study that revealed the recurrence of atrial tacky arrhythmia was significantly lower in the cryo‐balloon ablation group (42.9% vs. 67.8%, HR: 0.48 [95% CI, 0.35−0.66]; *p* < .001). It also showed that the effect of cryo‐balloon ablation on decreasing the recurrence of symptomatic atrial arrhythmia was even more (11% vs. 26.2%, HR: 0.39 [95% CI, 0.22−0.68]).^5^ The RAAFT‐2 study also demonstrated that AF patients who underwent radiofrequency ablation as first‐line treatment would less likely to experience the recurrence of symptomatic atrial arrhythmia compared to AAD therapy (HR, 0.56 [95% CI, 0.33−0.95]; *p* = .03) also the overall number of atrial arrhythmia episodes were significantly lower in ablation group (HR, 0.33 [95% CI, 0.28−0.4], *p* < .001).^22^ Seemingly, Poole et al. selected 56% of the CABANA population to study atrial arrhythmias recurrence utilizing a Trans‐Telephonic Monitor electrocardiogram monitoring system and demonstrated that ablation was effective in reducing any AF recurrence (by 48%) and reducing symptomatic AF (by 51%) in a total 60 months follow‐up.^13^ The study revealed that ablation significantly reduces the recurrence of symptomatic AF (HR: 0.49, [0.39−0.61]), time to the first recurrence of any AF (0.52, [0.45−0.6]), and time to first atrial arrhythmia recurrence (0.53, [0.46−0.62])^13^

Ablation also might cause a relative risk reduction of stroke/TIA compared to medical management as it could result in a better sinus rhythm maintenance and therefore lower risk of thrombosis due to lesser blood stasis in LA. The result of our study showed that the risk reduction of stroke/TIA was not significant in the ablation group. There were some confounding factors including assuming procedural TIAs without any significant complication with the same value of disabling stroke events, and another factor might be the inadequate follow‐up time in some of the trials for assessing long‐term AF complications like stroke or TIA. We could not eliminate the first factor due to lack of available information, but for minimizing the latter mentioned confounding factor, we decided to check the interventions' effect on stroke/TIA only in studies with a minimum follow‐up of 12 months, but again the result was not significant. Likewise, two different previous meta‐analyses done by Mao et al. and Asad et al. resulted in nonsignificant stroke/TIA risk reduction in the CA group compared to BMT (RR: 0.7, [0.39−1.23]) and (RR: 0.56, [0.26−1.22] respectively).^14^, ^15^ In contrast, Song et al. in their meta‐analysis declared that the effect of ablation on the risk of stroke/TIA in AF patients could be assumed to be significant (RR: 0.61, [0.39−0.96]) These results might be due to separating AF induced stroke from procedural ones.^16^

Analyses also showed that CA would result in a remarkable decrease in total hospitalization rate of AF patients compared to medical management but we could not have statistical analysis on cardiovascular or HF hospitalization (CVH or HFH), whereas some of our included studies showed a significant reduction in CVH, and HFH.^23^, ^24^ In agreement to our findings, CASTLE‐AF trial showed a significant lower HFH (HR: 0.56 [0.37−0.83]) and CVH (HR: 0.72 [0.52−0.99]) and even lesser cardiovascular death (0.49 [0.29−0.84]).^7^ CABANA also showed that death and CV hospitalization were somehow lower in the ablation group compared to medical therapy (HR: 0.83, [0.74−0.93], *p* = .001).^38^ These estimated effects of ablation had been influenced by treatment crossover (27.5% from BMT to ablation) and lower event rates than anticipated.^38^

The most considerable decrease in the rate of hospitalization in statistical analyses was noticed in the naive AF subgroup who has not received any treatment for AF before the trial. It could be a sign of better morbidity control and complication reduction by considering ablation as the first‐line treatment in PAF patients who are naive to therapy.

Our analyses for the first time suggested that compared to medical therapy, ablation could significantly reduce and delay the progression to persistent AF, so patients with AF might take benefit from the consideration of early ablation. Seemingly, the ATTEST study revealed that early ablation could be the superior management for delaying the progression of the disease, as the development of persistent AF/AT in the ablation group was 10 times lower (HR: 0.107 [0.024−0.47]; *p* = .0031) with a considerably lower rate of persistent AF (0.0% vs. 10.2%; *p* = .0002).^21^ Also, studies showed a higher ablation‐induced complication rate in older patients so it might be safer and more beneficial to consider ablation early in the younger AF population.^43^, ^44^, ^45^

The ablation of AF in patients with concomitant HF seems too very beneficial. AF and HF could form a vicious cycle as persistent tachycardia due to AF may result in arrhythmia‐induced cardiomyopathy,^46^, ^47^, ^48^ and HF is a risk factor for AF development and progression.^49^ Our study showed ablation cause a significant LVEF improvement compared to medical therapy which could cause better ventricular function.

Catheter ablation could be chosen as the first‐line AF management in HF patients as it could not only improve the patients' LVEF and ventricular function but also it may cause a reduction in hospitalization due to HF progression. There were also some other reviewed outcomes in several included studies which may be considered by the readers. CAPA study examined 648 patients with persistent or long‐standing persistent AF (327 ablations, 321 Best medical treatment) for 54 ± 10.6 months follow‐up and concluded that new‐onset congestive HF incidence is lower in the ablation group (2.8% vs. 7.2%, *p* < .001).^36^ CABANA, the vastest RCT on comparing ablation versus best medical therapy, had a subgroup of HF patients with AF, which 778 patients had been investigated in a 48.5‐month median follow‐up, which revealed that ablation is the superior therapy compared to BMT (rate control or AAD) according to lower primary outcome event (stroke, death, bleeding, cardiac arrest) (HR: 0.64 [95% CI, 0.41−0.99]), lower all‐cause mortality (relative reduction: 43%, HR: 0.7 [95% CI, 0.33−0.96]), better survival, and greater sinus rhythm maintenance in ablation group.^12^ The study also studied the results of patients in different subgroups according to their LVEF and showed that the effect of ablation on reduction in mortality was especially greater in patients with LVEF of 50% or more (HR: 0.4 [0.18−0.88]) compared to patients with LVEF of 40−49% (HR: 0.43 [0.09−2.13]).^12^ Another study on refractory AF patients who had HF with LVEF ≤ 35%, showed that catheter ablation of AF is attributed to significantly lower HFH (20.7% vs. 35.9%, HR: 0.56 [0.37−0.83], *p* = .004), cardiovascular hospitalization (HR: 0.72 [0.52−0.99]), cardiovascular death (11.2% vs. 22.3%, HR: 0.48 [0.29−0.84], *p* = .009), and all‐cause mortality (13.4% vs. 25%; HR: 0.53 [0.32−0.86], *p* = .01) in a median follow‐up of 37.8 months.^7^

This study has faced some limitations. Due to a lack of data on the distinction between noncomplicated procedural TIA and disabling stroke, we could not separate procedural TIA from stroke/TIA resulting from AF thromboembolic events. We tried analyses of studies with follow‐up of at least 12 months to minimize this confounding factor, but we could not remove these confounding factors' effect on our results. Lack of data on some of our subgroups made it impossible to do all the subgroup analyses completely. We were supposed to have another subgroup analysis on comparing the effect of crayon‐balloon ablation versus radiofrequency catheter ablation as two different methods of ablation in AF patients and see which one is more effective, but as cryo‐balloon was used just in trials assessing AF patients who were naïve to AAD therapy and RF CA population was not the same (refractory AF to AAD, persistent AF, PAF with HF), the combination and comparing these two subgroups became unreasonable and impossible. Publication bias tests resulted positive in the analysis of AF recurrence, it could be due to the high‐quality and legitimate included RCTs.^50^ The current study was limited by the lack of data on different causes of mortality as we could not separate mortalities due to cardiovascular causes or AF complications from other causes.

Our current meta‐analyses revealed the valuable beneficial effects of Ablation on decreasing mortality, hospitalization, stroke/TIA, AF recurrence (also any atrial arrhythmia recurrence), as well as improving LVEF and postponing the AF progression in different stages of AF, signifying the superiority of catheter ablation to medical management in patients with AF (Tables 1, 2).

**Table 1:** **Characteristics of the included randomized controlled trials**.

Study	Year	Population	Intervention/comparison	Sex, male (%)	Age	Sample size	Echo parameters		NYHA class ≥ 2 (%)	BMI	Follow‐up
							LAD	LVEF			
Kuniss et al.	2021	Treatment Naïve Symptomatic recurrent PAF	Cryo‐balloon CA	71.0	50.5 (13.1)	107	37 (5.9) −46.8 (8.2)	62.8 (5.4)			12
			AAD class 1/3	64.9	54.1 (13.4)	111	38 (4.9)−47.7 (6.3)	63.7 (5.4)			
Poole et al. (CABANA)	2021	Symptomatic AF patients ≥ 65 year old or <65 year old with ≥1 risk factor for stroke (Sample of CABANA: 1240)	PVI RF CA ± additional ablation	65.5	Median (Q1, Q3) 68 (64, 73)	611		EF ≤ 35: 5.5%	26.6	(Median) 31	60
			AAD	65.7	68 (64, 73)	629		EF ≤ 35: 2.7%	26.9	30	
Packer et al. (CABANA)	2019	Symptomatic AF patients ≥ 65 year old or <65 year old with ≥1 risk factor for stroke	PVI RF CA ± additional ablation	62.7	67 (7.4)	1108		EF ≤ 35: 4.8%	34.3	Median (Q1−Q3) 30 (27−34)	60
			AAD and/or MRC	63	67 (7.4)	1096		EF ≤ 35: 4.2%	36.7	30 (26−35)	
Packer et al. (CABANA)	2021	NYHA class ≥ 2 in CABANA population	PVI RF CA ± additional ablation	54.8	68	378			100	31	60
			AAD and/or MRC	56.5	67	400			100	31	
Wu et al. (CAPA study)	2020	Persistent and long‐standing AF (648)	RF CA	66.7	64.8 (12.6)	327	45 (8.5)	53.3 (9.3)			54.2 ± 10.6
			AAD class 1 or 3 ± electric cardioversion	63.2	64.4 (13.6)	321	46 (7.8)	51.9 (9.4)			
Andrade et al.	2020	Symptomatic, paroxysmal untreated AF (303)	Cryo‐balloon ablation	72.7	57.7 (12.3)	154	39.5 (5)	59.6 (7)	9.1	30.9 (14.2)	12
			AAD class 1 or 3	68.5	59.5 (10.6)	149	38.1(6.5)	59.8 (7.6)	9.4	29.7 (9.3)	
Wazni et al.	2020	Paroxysmal AF naïve to treatment	PVI with Cryo‐balloon CA	61	60.4 (11.2)	104	38.7 (5.7)	60.9 (6)			12
			AAD class 1 or 3	58	61.6 (11.2)	99	38.2 (5.4)	61.1 (5.9)			
Kuck et al. (ATTEST)	2020	Paroxysmal AF ≥ 60 y/o for ≥ 2 years and with ≥ 2episodes over the 6 months preceding enrollment	PVI via RF CA	42.2	67.8 (4.8)	102	42.1 (6.1)	61.8 (5.8)			36
			AAD class 1 or 3	41.7	67.6 (4.6)	123	43.4 (5.6)	62.3 (5.2)			
Kuck et al. (AMICA)	2019	Persistent or long standing persistent AF and LVEF ≤ 35, have indication for ICD or CRT‐D	CA ± drug therapy ± device	60	65 (8)	68	50 (6)	27.8 (9.5)	100	29.4 (5)	12
			Rhythm or rate control ± device	66	65 (8)	72	51 (5)	24.8 (8.8)	100	28.4 (4.5)	
			MRC ± defibrillator	51	74 (9)	70			100		
Sugumar et al. (CAMERA ‐MRI)	2020	Persistent AF with idiopathic cardiomyopathy (LVEF ≤ 45%)	PVI and PWI RF CA	96.7	58.8 (11.8)	33	32.6 (7.4)	31.7 (9.8)		30.4 (7.7)	48
			MRC	93.3	65.5 (7.2)	33	35.2 (5.5)	31.5 (8.4)		29 (3.1)	
Prabhu et al. (CAMERA ‐MRI)	2017	Persistent AF with idiopathic cardiomyopathy (LVEF ≤ 45%)	PVI and PWI RF CA	94	59 (11)	33	48 (5.5)	32 (9.4)		30 (7.5)	6
			MRC	88	62 (9.4)	33	47 (8.2)	34 (7.8)		31 (4.1)	
Packer et al. (STOP AF)	2013	Symptomatic paroxysmal AF, previously failed therapy with ≥1 membrane active AAD	Cryo‐balloon CA	76.7	57 (9)	163	40 (5)	60 (6)	6.7		12
			AAD	78	56 (9)	82	41 (6)	61 (6)	6.1		
Pappone et al. (APAF)	2006	Patients with paroxysmal AF of 6 ± 5 years duration who failed AAD therapy, age 56 ± 10 years (198)	Circumferential PVI ablation (RF CA)	69.7	55 (10)	99	40 (6)	60 (8)			12
			AAD (maximum tolerable class 1 or 3)	64.6	57 (10)	99	38 (6)	61 (6)			
Pappone et al. (APAF)	2011	Patients with paroxysmal AF of 6 ± 5 years duration who failed AAD therapy, age 56 ± 10 years (198)	Circumferential PVI ablation (RF CA)	69.7	55 (10)	99	40 (6)	60 (8)			48
			AAD (maximum tolerable class 1 or 3)	64.6	57 (10)	99	38 (6)	61 (6)			
Marrouche et al. (CASTLE‐AF)	2018	Heart failure patients with symptomatic paroxysmal or persistent AF who failed response to AAD, unacceptable side effects or unwillingness to take AAD, who have ICD or CRT‐D	PVI RF CA	87	Median (IQR) 64 (56−71)	179	Median (IQR) 48 (45−54)	Median (IQR) 32.5 (25−38)	89		60
			Rate or rhythm control	84	64 (56−73.5)	184	49.5 (45−55)	31.5 (27−37)	89		
Di Biase et al. (AATAC)	2016	Heart failure patients with persistent AF with dual chamber ICD or CRT‐D	RF CA	75	62 (10)	102	47 (4.2)	29 (5)	100	30 (8)	24
			AAD	73	60 (11)	101	48 (4.9)	30 (8)	100	29 (4)	
Sohara et al.	2016	Symptomatic paroxysmal AF refractory to AAD (class 1 to 4)	Hot Balloon ablation PVI RFA	80	59 (10)	100	38.3 (6)	66.7 (6)			9
			AAD	81	61 (10)	43	38.3 (5)	66.5 (7)			
Morillo et al. (RAAFT‐2)	2014	Treatment‐naïve patients with paroxysmal AF	RF CA	77.3	56.3 (9.3)	66	40 (5)	61.4 (4.8)			24
			AAD	73.8	54.3 (11.7)	61	43 (5)	60.8 (7)			
Mont et al. (SARA)	2013	Persistent AF (excluding long‐standing persistent AF)	RF CA	77.5	55 (9)	98	41.3 (4.6)	61.1 (8.8)	25.5		12
			AAD class 1c or 3	77	55 (9)	48	42.7 (5.1)	60.8 (9.7)	18.8		
Hunter et al. (CAMTAF)	2014	Symptomatic heart failure patients with LV systolic dysfunction and persistent AF	RF CA	96.2	55 (12)	26	52 (11)	31.8 (7.7)	100		6‐12
			MRC	95.8	60 (10)	24	50 (10)	33.7 (12.1)	100		
Hummel et al. (TTOP‐AF)	2014	Persistent or long‐standing persistent AF who had failed at least one AAD	RF CA	83.3	59.6 (8.3)	138	45 (5)	54.7 (7.1)			6
			AAD	83.3	60.7 (8.9)	72	46 (5)	54.9 (6.7)			
Pokushalov et al.	2013	Symptomatic PAF after a previously failed RF ablation	RF CA	73	56 (7)	77	45 (7)	57 (6)			12
			AAD	77	57 (7)	77	46 (5)	58 (5)			
Jones et al.	2012	Adults with symptomatic heart failure, radionucleotide LVEF ≤ 35%, and persistent AF	RF CA	81	64 (10)	26	50 (6)	22 (8)	100		12
			MRC	92	62 (9)	26	46 (7)	25 (7)	100		
Nielsen et al.	2012	Paroxysmal AF who are naïve to AAD treatment	RF CA	68	56 (9)	146	40 (6)	‐	10.3	27 (4)	24
			AAD	72	54 (10)	148	40 (5)	‐	13.5	27 (4)	
MacDonald et al.	2010	Patients with advanced heart failure, severe LV dysfunction, and persistent AF	RF CA	77	62.3 (6.7)	22	‐	16.1 (7.1)	100	30 (5.7)	6
			MRC	79	64.4 (8.3)	19	‐	19.6 (5.5)	100	30 (5.6)	
Wilber et al.	2010	Symptomatic paroxysmal AF who did not respond to at least 1 AAD, who experienced ≥ 3 AF episodes within 6 months before randomization	RF CA	68.9	56 (9)	106	40 (1.1)	62.3 (2)			9
			AAD class 1 or 3	62	56 (13)	61	40.5 (1.5)	62.7 (2)			
Forleo et al.	2008	Patients with DM type 2 and paroxysmal or persistent AF	RF CA	57.1	63.2 (8.6)	35	44.3 (5.6)	54.6 (7)			12
			AAD	65.7	64.8 (6.5)	35	45.2 (5.2)	52.6 (8.6)			
Jais et al.	2008	Paroxysmal AF resistant to at least 1 AAD	PVI RF CA	84.9	49.7 (10.7)	53	39.5 (5.6)	63.1 (11)			12
			AAD	83.1	52.4 (11.4)	59	40 (5.7)	65.6 (7.2)			
Stabile et al.	2005	Paroxysmal or persistent AF who were intolerant of AAD or in whom ≥2 AAD regimens had failed	RF CA + AAD	54.4	62.2 (9)	68	46 (5)	59.1 (6.7)			12
			AAD alone	63.8	62.3 (10.7)	69	45.4 (5.5)	57.9 (5.8)			
0ral et al.	2006	Chronic AF	Circumferential PVI RF CA	87	55 (9)	77	45 (6)	55 (7)			12
			AAD	92.5	58 (8)	69	45 (5)	56 (7)			
Wazni et al.	2005	AAD‐naïve patients who experienced monthly symptomatic AF episodes for at least 3 months	PVI RF CA	‐	53 (8)	33	41 (8)	53 (5)			12
			AAD	‐	54 (8)	37	42 (7)	54 (6)			

Abbreviations: AAD, antiarrhythmic drugs; AF, atrial fibrillation; BMI, body mass index; CA, catheter ablation; CRT‐D, cardiac resynchronization therapy defibrillator; DM, diabetes mellitus; ICD, implantable cardioverter defibrillator; IQR, interquartile range; LAD, left atrial diameter; LV, left ventricle; LVEF, left ventricular ejection fraction; MRC, medical rate control; NYHA, New York Heart Association functional class of heart failure; PAF, paroxysmal atrial fibrillation; PVI, pulmonary vein isolation; PWI, posterior wall isolation; RF, radiofrequency; RF CA, radiofrequency catheter ablation.

**Table 2:** **Results of analysis of articles in which compared the effect of ablation versus medical treatment for patients with AF** (Have been illustrated in Supporting Information S1: Figure 2−18 in Supporting Information Materials.).

Analysis	Subgroups	N. studies	RR (95% CI)^a^
SAE	All	10	0.92 [0.64−1.33]
	Cryo‐balloon ablation	4	0.78 [0.34−1.78]
	Radiofrequency ablation	6	1.02 [0.57−1.82]
AA recurrence	All	20	0.46 [0.39−0.54]
	Cryo‐balloon ablation	4	0.44 [0.29−0.66]
	Radiofrequency ablation	16	0.46 [0.38−0.56]
	Naïve	6	0.53 [0.37−0.76]
	Refractory	9	0.39 [0.30−0.50]
	Paroxysmal	8	0.47 [0.35−0.64]
	Persistent	3	0.43 [0.18−1.01]
AF recurrence	All	12	0.48 [0.38−0.61]
	Naïve	4	0.52 [0.25−1.07]
	Refractory	5	0.40 [0.24−0.70]
	Persistent	3	0.46 [0.32−0.66]
Hospitalization	All	9	0.43 [0.27−0.67]
	Cryo‐balloon ablation	2	0.30 [0.07−1.35]
	Radiofrequency ablation	7	0.45 [0.24−0.86]
	Naïve	3	0.28 [0.14−0.54]
	Refractory	4	0.35 [0.25−0.47]
	Paroxysmal	5	0.32 [0.23−0.46]
Stroke/TIA	All	12	0.71 [0.44−1.14]
	Paroxysmal	10	0.77 [0.44−1.34]
	Persistent	4	0.80 [0.24−2.64]
	Follow‐up ≥ 12 months	8	0.62 [0.37−1.04]
Mortality	All	11	0.71 [0.57−0.88]
	Refractory	3	0.53 [0.06−4.53]
	Persistent	5	0.69 [0.37−1.30]
	Heart failure	4	0.64 [0.27−1.51]
Progression to persistent AF	All	3	0.11 [0.02−0.65]
Echography indexes	Subgroups	N. studies	(mean difference [95% CI])^b^
LVEF change	All	8	6.84 [3.27−10.42]
	Persistent	7	6.39 [2.26−10.53]
	Heart failure	7	6.39 [2.26−10.53]

Abbreviations: AA, atrial arrhythmia; AF, atrial fibrillation; CI, confidence interval; HF, heart failure; LAD, left atrial diameter; LVEF, left ventricular ejection fraction; N., number of; RR, risk ratio; SAE, serious adverse events; TIA, transient ischemic attack.

^a^ Risk ratio less than 1 indicates lower relative risk of each event in ablation groups and higher than 1 indicates a higher risk of that event in medical therapy patients;

^b^ positive mean difference indicates higher improvement of the echocardiographic index in the ablation group and a negative mean difference indicates higher index decrement in the ablation group compared to medical therapy.

## AUTHOR CONTRIBUTIONS

According to ICMJE, all authors accomplish the authorship criteria. All authors got involved similarly in writing the draft and emerging the idea. Fatemeh Kheshti, Alireza Hosseinpour, Mehdi Bazrafshan, Saeed Abdollahifard, and Armin Attar contributed to the search, screening process, and analyzing data. Lastly Armin Attar, Saeed Abdollahifard, and Fatemeh Kheshti revised the final draft.

## CONFLICT OF INTEREST STATEMENT

The author declare no conflict of interest.

## Supporting information

Supporting information.

## Data Availability

The data that support the findings of this study would be available upon reasonable request from the corresponding author.
