# Supplementary material for: Ablation versus medical therapy for patients with atrial fibrillation: An updated meta‐analysis
Source: Clin Cardiol. 2023 Nov 8;47(2):e24184. doi: 10.1002/clc.24184 (PMC10826237; doi:10.1002/clc.24184)
Supplement: Supplementary file 1 — Supporting information. [file CLC-47-e24184-s001.docx]

**Supplementary material**

**Part A: Search strategy**

**Part B: Risk of bias and quality assessment**

**Part C: A more detailed table of Outcomes of included studies**

**Part D: Forest plots of analysis**

**Part A: Search strategy**

(((Ablation, Radiofrequency[tiab] OR Radio Frequency Ablation[tiab] OR Ablation, Radio Frequency[tiab] OR Radio-Frequency Ablation[tiab] OR Ablation, Radio-Frequency[tiab] OR Radiofrequency Ablation[MeSH Terms] OR Cryosurgery[MeSH Terms] OR Cryosurgeries[tiab] OR Cryoablation[tiab] OR Cryoablations[tiab] OR Catheter Ablation[MeSH Terms] OR Ablation, Catheter[tiab] OR Catheter Ablation, Transvenous[tiab] OR Transvenous Catheter Ablation[tiab] OR Ablation, Transvenous Catheter[tiab] OR Catheter Ablation, Electric[tiab] OR Electrical Catheter Ablation[tiab] OR Catheter Ablation, Electrical[tiab] OR Ablation, Electrical Catheter[tiab] OR Electric Catheter Ablation[tiab] OR Ablation, Electric Catheter[tiab] OR Ablation, Transvenous Electric[tiab] OR Electric Ablation, Transvenous[tiab] OR Transvenous Electric Ablation[tiab] OR Ablation, Transvenous Electrical[tiab] OR Electrical Ablation, Transvenous[tiab] OR Transvenous Electrical Ablation[tiab] OR Catheter Ablation, Radiofrequency[tiab] OR Radiofrequency Catheter Ablation[tiab] OR Ablation, Radiofrequency Catheter[tiab] OR Catheter Ablation, Percutaneous[tiab] OR Percutaneous Catheter Ablation[tiab] OR Ablation, Percutaneous Catheter[tiab] OR ablation[tiab] )) AND ((Atrial Fibrillation[MeSH Terms] OR Atrial Fibrillations[tiab] OR Fibrillation, Atrial[tiab] OR Fibrillations, Atrial[tiab] OR Auricular Fibrillation[tiab] OR Auricular Fibrillations[tiab] OR Fibrillation, Auricular[tiab] OR Fibrillations, Auricular[tiab] OR Persistent Atrial Fibrillation[tiab] OR Atrial Fibrillation, Persistent[tiab] OR Atrial Fibrillations, Persistent[tiab] OR Fibrillation, Persistent Atrial[tiab] OR Fibrillations, Persistent Atrial[tiab] OR Persistent Atrial Fibrillations[tiab] OR Familial Atrial Fibrillation[tiab] OR Atrial Fibrillation, Familial[tiab] OR Atrial Fibrillations, Familial[tiab] OR Familial Atrial Fibrillations[tiab] OR Fibrillation, Familial Atrial[tiab] OR Fibrillations, Familial Atrial[tiab] OR Paroxysmal Atrial Fibrillation[tiab] OR Atrial Fibrillation, Paroxysmal[tiab] OR Atrial Fibrillations, Paroxysmal[tiab] OR Fibrillation, Paroxysmal Atrial[tiab] OR Fibrillations, Paroxysmal Atrial[tiab] OR Paroxysmal Atrial Fibrillations[tiab]))) AND ((best medical treatment[tiab] OR medical treatment[tiab] OR BMT[tiab] OR medical management[tiab] OR drug therapy[tiab] OR medical[tiab] OR best medical therapy[tiab] OR Antiarrythmic drug [tiab] OR rate control drug[tiab] OR medication[tiab] OR drug[tiab] OR pharmacotherapy [tiab] OR Antiarrythmic medication [tiab] OR rate control medication [tiab])

**Part B: Risk of bias and quality assessment**

*
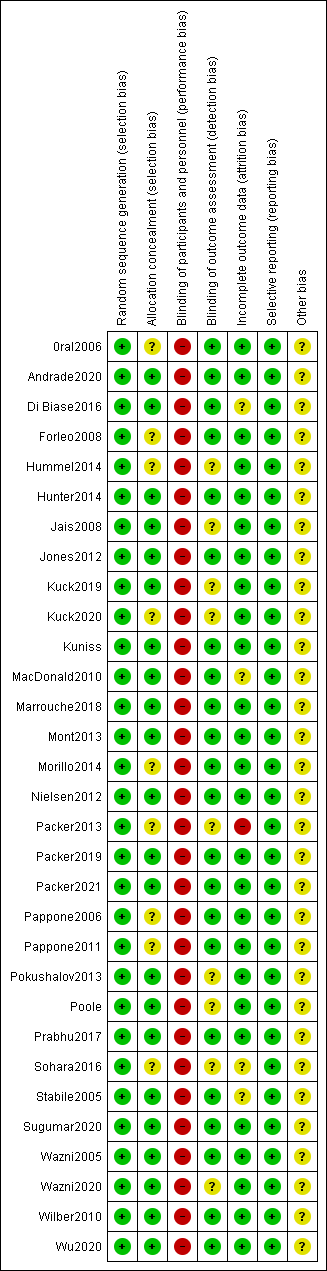
*

**Figure s1.** *Risk of bias summary: review authors' judgements about each risk of bias item for each included study.*

**Part C: A more detailed table of Outcomes of included studies**

**Table 1: Outcome table of included studies investigating the effect Ablation versus medical treatment in management of AF patients**

| **Study** | **Sub groups** | | **mortality** | **Stroke or TIA** | **LVEF change** | **LAD change** | **Atrial Arrhythmia recurrence** | **AF**  **recurrence** | **Hospitalization** | **Crossover**  **(%)** |
| --- | --- | --- | --- | --- | --- | --- | --- | --- | --- | --- |
|  |  |  | **% A/BMT**  **HR (95%CI)** | **% A/BMT**  **HR (95%CI)** | **MD±SD** | **MD±SD**  **(cm^2^)** | **% A/BMT**  **HR (95%CI)** | **% A/BMT**  **HR (95%CI)** | **Events A/BMT** | **(%)** |
| Kuniss et al. |  | 0/0 | | 0/0 |  |  | 17.8 / 32.4 | 11.21 / 20.72 | 0.67 (0.41-1.10)* | 9 |
| Poole et al. |  |  | |  |  |  | 51.8/70.58  0.53 (0.46-0.62) | 49.91 / 69.4  0.52(0.45-0.6) |  |  |
| Packer et al. (CABANA) 2019 |  | 5.2 / 6.1  0.85 (0.60-1.21) | | 0.3 / 0.6  0.42(0.11-1.62) |  |  | 0.53(0.46-0.62) | 0.52(0.45-60) |  | 27.5 |
| Packer et al. (CABANA) 2021 | All | 0.57 (0.33-0.96) | |  |  |  |  | 0.56(0.42-0.74) |  | 22.3 |
|  | EF≥50 | 0.4 (0.18-0.88) | |  |  |  |  |  |  |  |
|  | EF: 40-49 | 0.43 (0.09-2.13) | |  |  |  |  |  |  |  |
| Wu et al.  (CAPA study) | ALL | 1.5 / 1.6 | | 4.2 / 7.2  0.57 (0.42-0.79) |  |  |  | 29.7 / 71 |  | 0 |
|  | Persistent |  |  |  |  |  |  | 14.6 /52.3 |  |  |
|  | LS PAF |  |  |  |  |  |  | 40.5 / 83.8 |  |  |
| Andrade  et al. |  | 0/0 | | 0 / 0.67% |  |  | 33.8 / 67.8  0.38(0.27-0.53) |  | 3.2 / 8.7  0.37 (0.14, 1.02)** | 24.2 |
| Wazni et al. 2021 |  |  | |  |  |  |  | 20.2 / 35.4 | 13 / 44 | 34.3 |
| Kuck et al. (ATTEST) |  |  | |  |  |  | 49.2 / 84.8 |  |  | 12.2 |
| Kuck et al. (AMICA) |  | 8.1 / 8 | |  | +8.8 [5.8-11.9] *** / +7.3 [4.3-10.3] |  |  |  |  | 4 |
| Sugumar et al. |  | 7.8 / 0 | |  | 16.4±13.3 / 8.6±7.6 | -4.8±7.5 / 2.3±2.7 |  |  | 0.6±1.2 / 0.7±0.8 | 54.5% |
| Prabhu et al. |  |  | |  | 18±13 / 4.4±13 | -12±13 / 1.7±14 **** |  |  |  | 9.1% |
| Packer et al. (STOP AF) |  |  | | 4.3 / 0 |  |  | 30 / 92.7 |  |  | 79% |
| Pappone et al. 2006 |  |  | |  |  |  |  | 11.1 / 75.8 | 9 / 167 | 42.4 |
| Pappone et al. 2011 |  |  | |  |  |  | 27.3 / 89.9 |  | 61 / 325 | 87.9 |
| Marrouche et al. |  | 13.4 / 25  0.53 (0.32-0.86) | | 2.8 / 6  0.46 (0.16-1.33) | +8 [2.2, 19.1] / +0.2 [-3, 16.1] ***** | -1 [-5, 6] / 0 [-5, 5]***** |  |  | 114 / 122 | 9.8 |
| Di Biase et al. |  | 8 / 18 | |  | 8.1 ± 4 / 6.2 ±5 |  |  | 30.4 / 62.6 | 32 / 58 |  |
| Sohara et al. |  |  | | 1.5 / 0 |  |  |  | 40 / 90.7  0.21(0.13-0.43) |  | 79.1 |
| Morillo et al. |  | 0/0 | | 0/0 |  |  | 47 / 59  0.56 (0.35-0.9) | 41 / 57  0.52(0.3-0.89) |  | 47.9 |
| Mont et al. |  |  | |  |  |  |  | 39.8 / 70.8 | 2 / 6.25 | 47.9 |
| Hunter et al. |  | 0 / 4.2 | | 3.8 / 0 | +8.1±13.3 / -3.6 ±10.6 |  |  |  |  |  |
| Hummel et al. |  | 0.7 / 0 | | 2.9 / 0 | +3.6 / +2.1 |  |  |  |  | 65 |
| Pokushalov et al. |  |  | |  |  |  | 41.6 / 88.3 |  |  | 56 |
| Jones et al |  |  | |  | 10.9±11.5 / 5.4±8.5% | -6.22 [-7.23, -2.68] / -2.62 [-5.99, 0.74] |  |  |  |  |
| Nielsen et al. |  | 2 / 2.7 | | 1.4 / 0.7 |  |  |  | 15 / 29  0.79(0.57-1.09) |  | 36 |
| McDonald et al. |  |  | | 4.54 / 0 | +8.2±12 / +1.4±5.9 |  | 50 / 100 |  |  |  |
| Wilber et al. |  |  | |  |  |  | 27 / 83  0.29 (0.18-0.45) |  |  | 64.3 |
| Forleo et al. |  |  | |  |  |  |  | 20 / 57.1 | 8.6 / 34.3 |  |
| Jais et al. |  | 0 / 3.38 | |  |  |  |  | 11 / 77 |  | 63 |
| Stabile et al. |  | 1.4 / 2.8 | | 1.4 / 0 |  |  | 44.1 / 91.3 | 38.2 / 91.3 |  | 57.1 |
| 0ral et al. |  |  | |  |  |  | 26 / 42 |  |  | 77 |
| Wazni et al. 2005 |  |  | |  |  |  |  | 13 / 63 | 3 / 19 |  |

*IRR (95%CI)

**RR (95%CI)

***[95%CI]

****ml/m^2^

*****IQR

A: ablation, BMT: best medical treatment, TIA: transient ischemic attack, LVEF: left ventricular ejection fraction, LAD: left atrial diameter, AF: atrial fibrillation, AAD: antiarrhythmic drugs, HR: hazard ratio, LS PAF: long standing persistent atrial fibrillation, IRR: incidence rate ratio, RR: risk ratio, MD: mean difference, SD: standard deviation, EF: ejection fraction, IQR: interquartile range

**Part D: Forest plots of analysis**

*Atrial fibrillation recurrence*

*
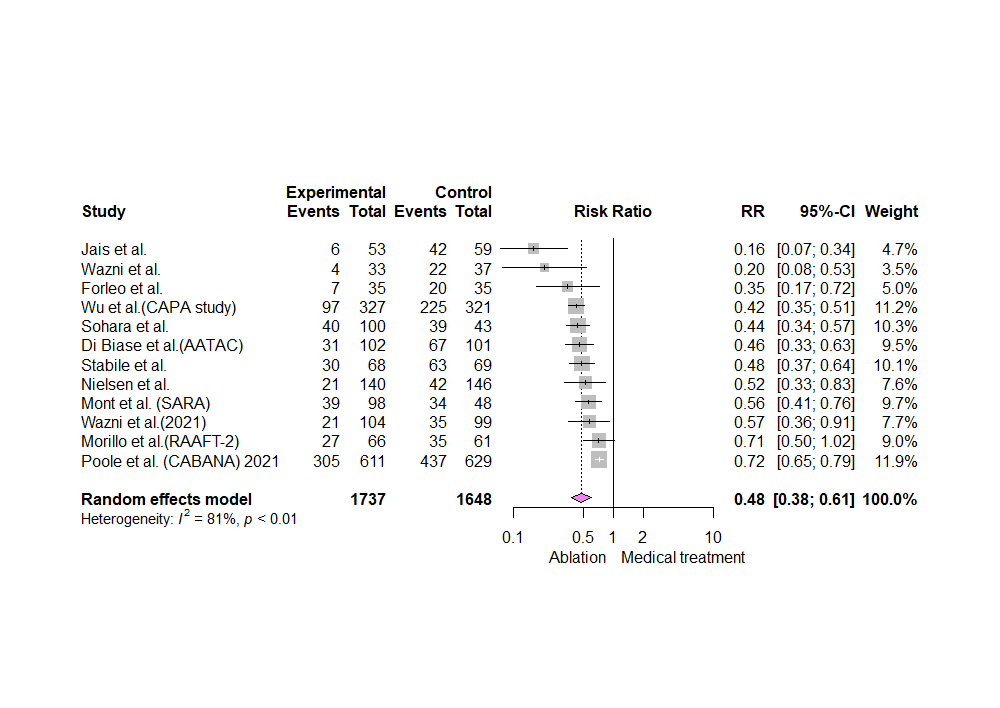
*

**Figure s2.** Forest plot of studies comparing Ablation versus medical therapy in term of AF recurrence in AF patients

*
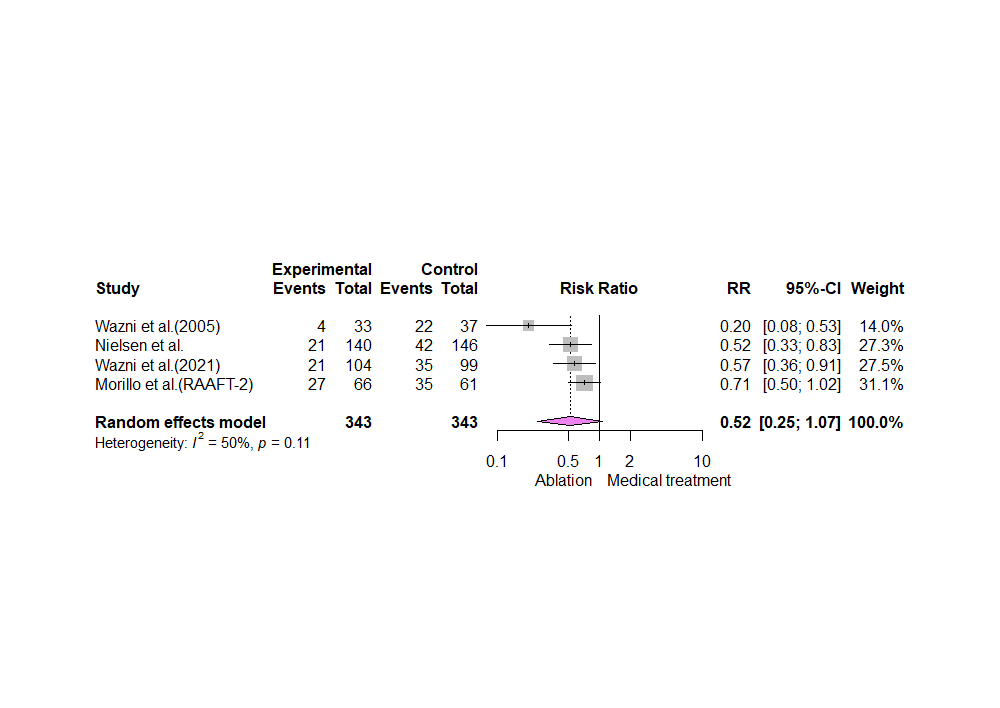
*

**Figure s3.** Forest plot of studies comparing Ablation versus medical therapy in term of AF recurrence in naïve AF subgroup

*
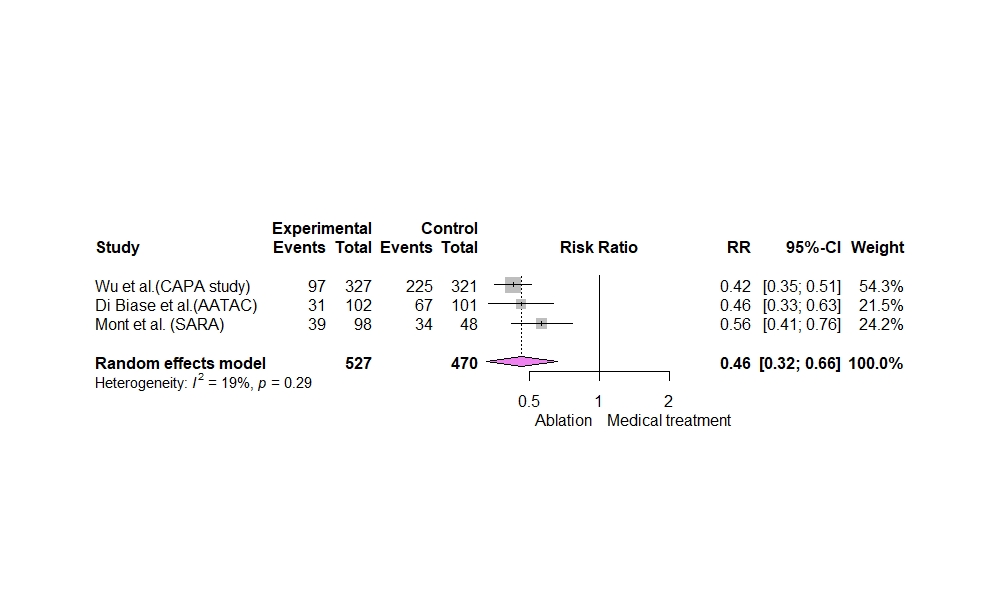
*

**Figure s4.** Forest plot of studies comparing Ablation versus medical therapy in term of AF recurrence in persistent AF subgroup

*
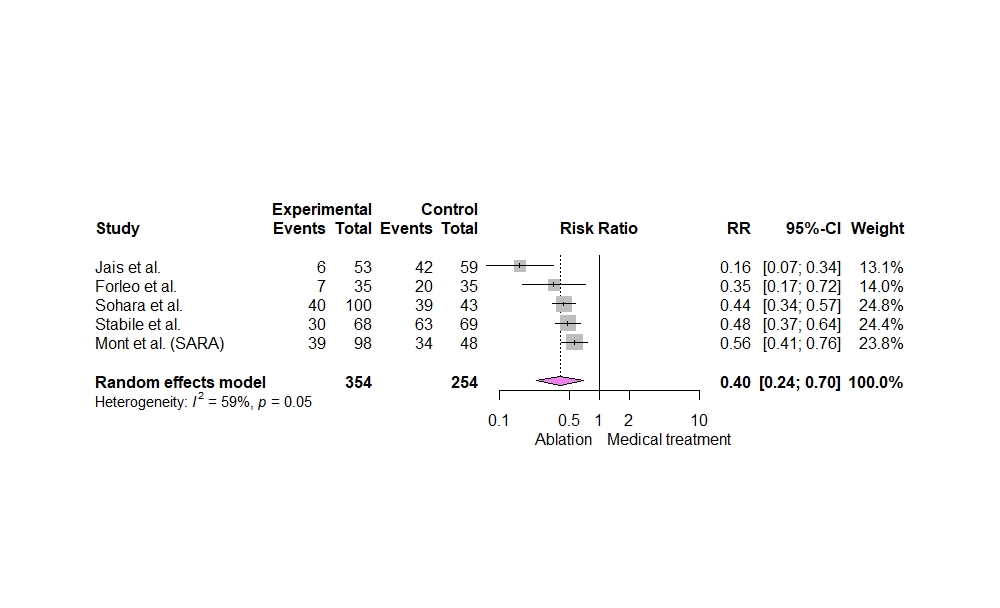
*

**Figure s5.** Forest plot of studies comparing Ablation versus medical therapy in term of AF recurrence in refractory AF subgroup

*Stroke/TIA*

*
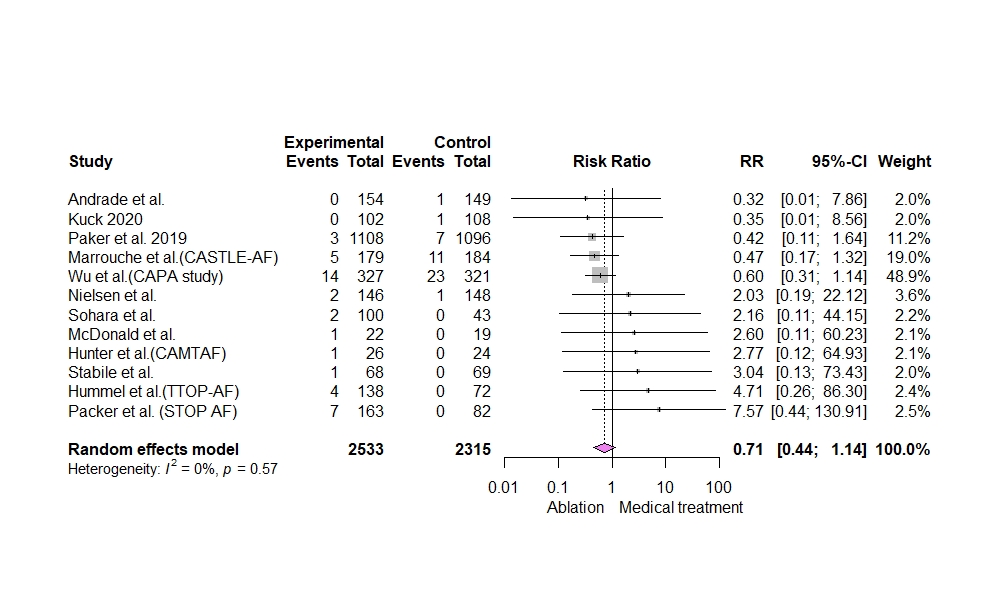
*

**Figure s6.** Forest plot of all included studies comparing Ablation versus medical therapy in term of Stroke/TIA event in AF

*
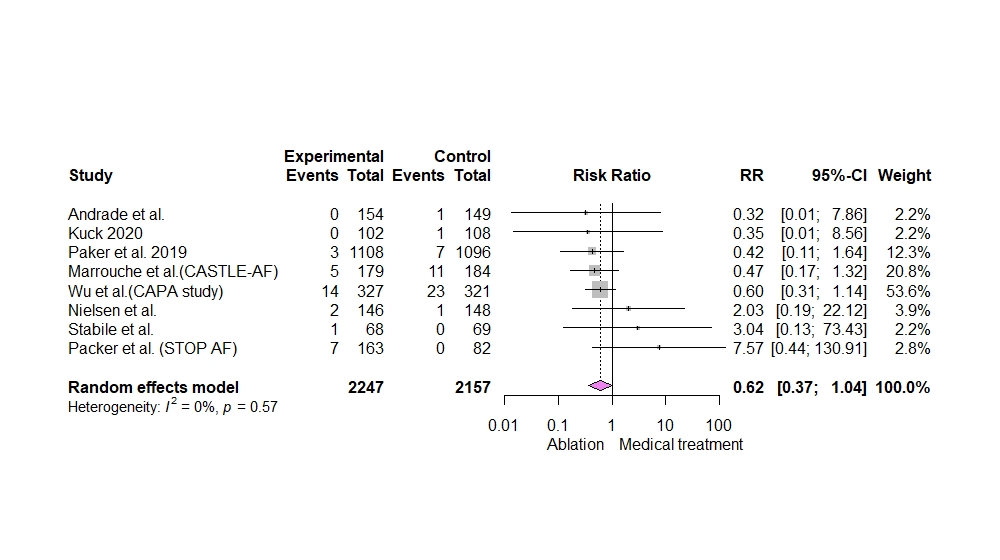
* **Figure s7.** Forest plot of studies with follow up period of at least 12 months comparing Ablation versus medical therapy in term of Stroke/TIA event in AF patients

*
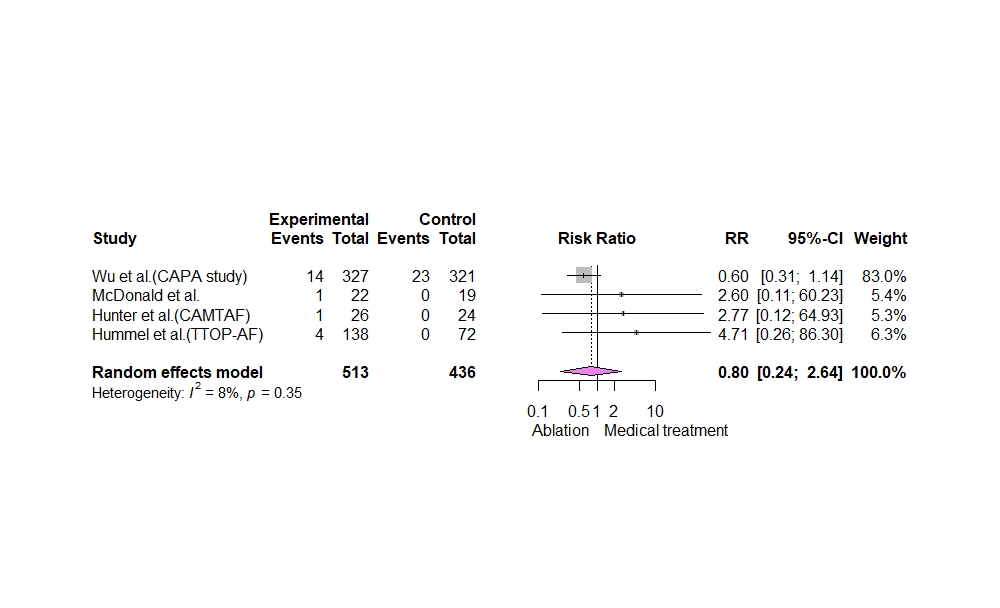
*

**Figure s8.** Forest plot of studies comparing Ablation versus medical therapy in term of Stroke/TIA event in persistent AF subgroup

*
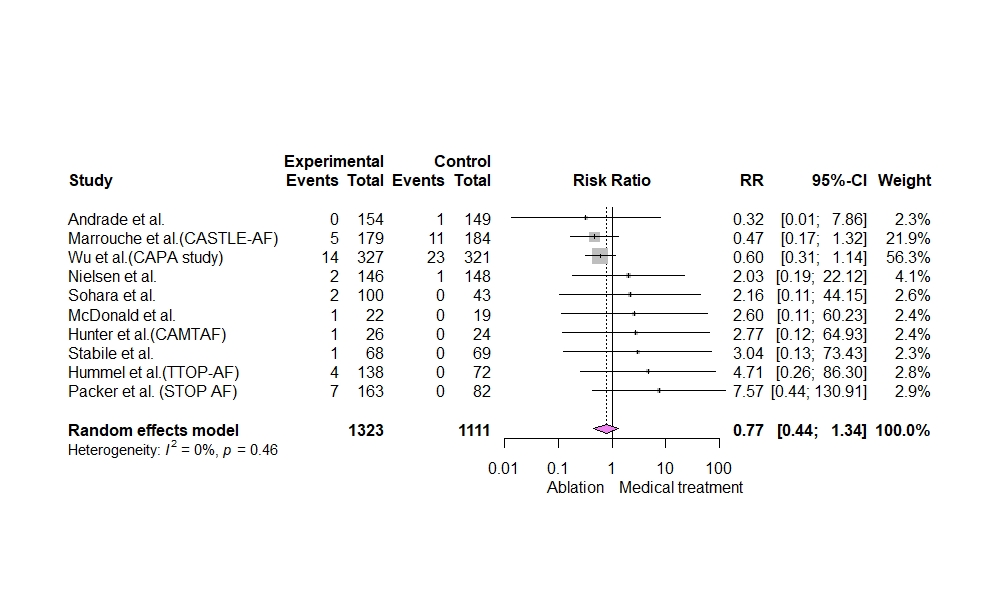
*

**Figure s9.** Forest plot of studies comparing Ablation versus medical therapy in term of Stroke/TIA event in paroxysmal AF subgroup

*Mortality*

*
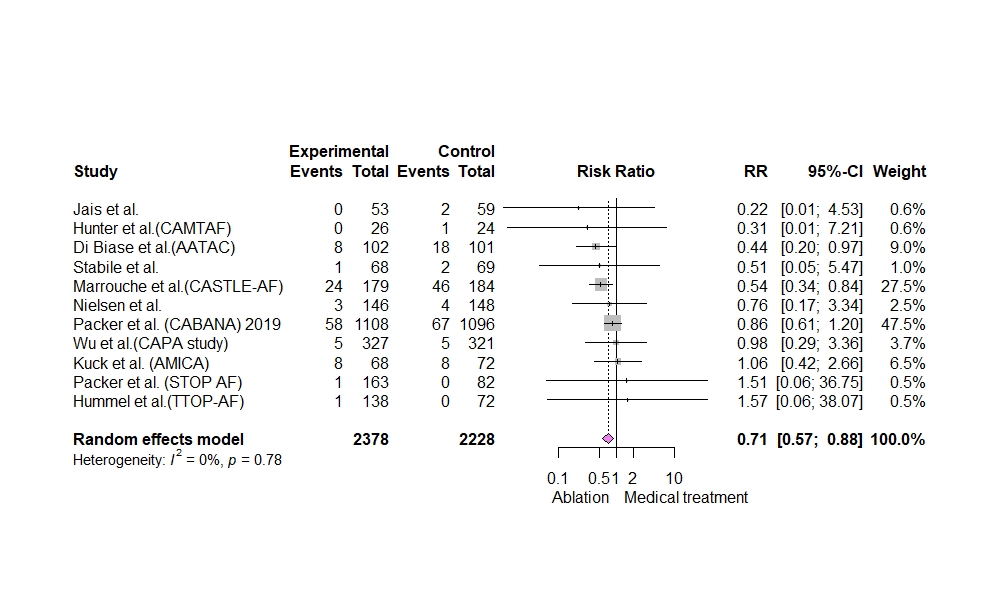
*

**Figure s10.** Forest plot of studies comparing Ablation versus medical therapy in term of mortality in AF patients

*
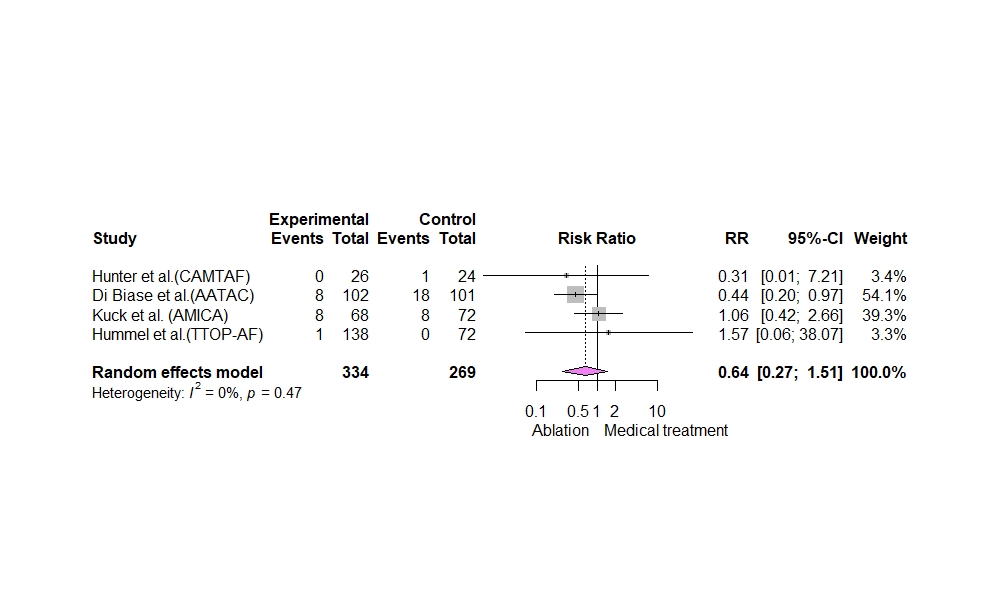
* **Figure s11.** Forest plot of studies comparing Ablation versus medical therapy in term of mortality in heart failure subgroup of patients with AF

*
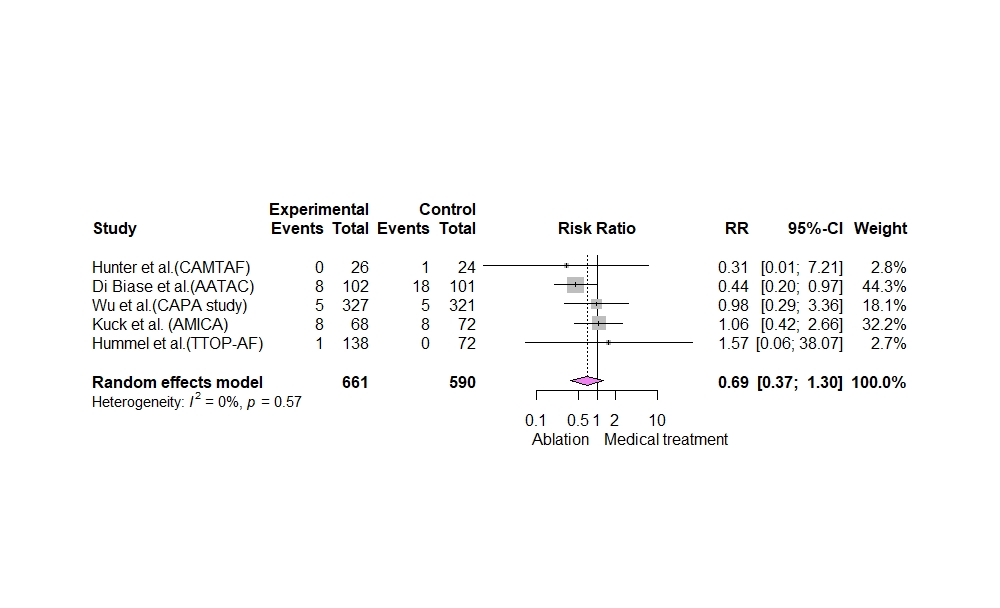
*

**Figure s12.** Forest plot of studies comparing Ablation versus medical therapy in term of mortality in persistent AF subgroup

*
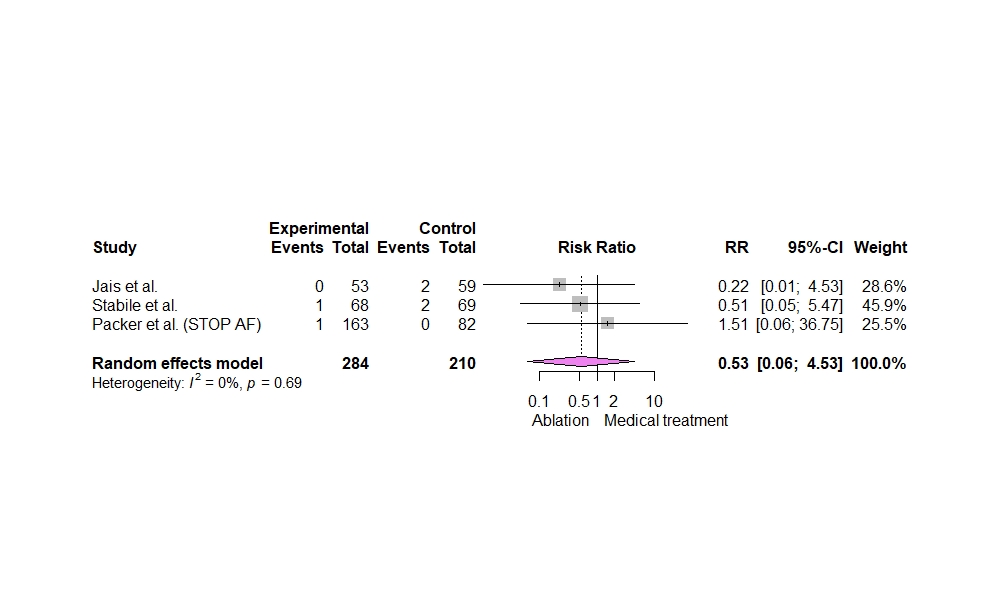
*

**Figure s13.** Forest plot of studies comparing Ablation versus medical therapy in term of mortality in refractory AF subgroup

*Hospitalization*

*
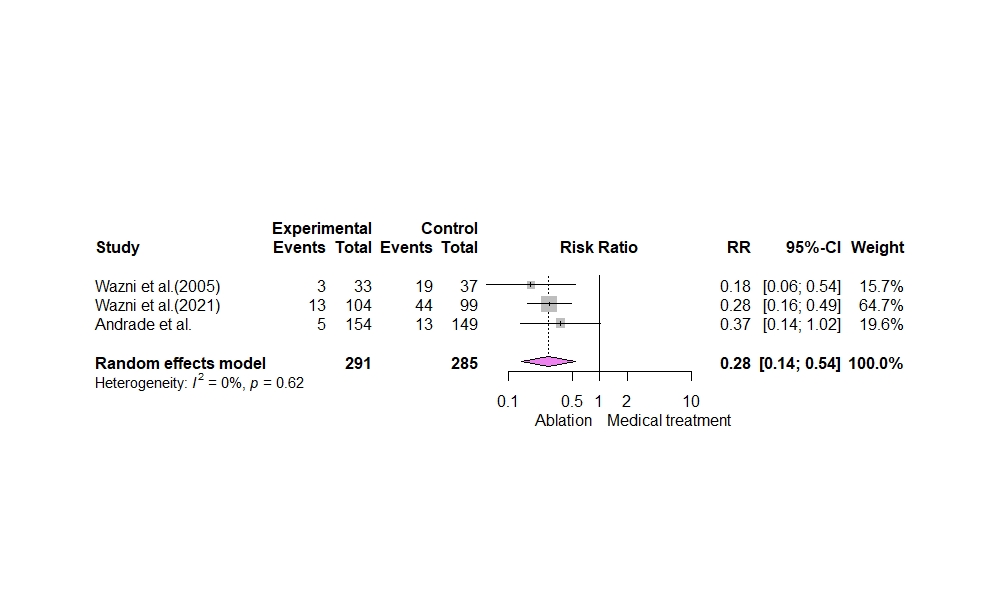
*

**Figure s14.** Forest plot of studies comparing Ablation versus medical therapy in term of hospitalization events in naïve AF subgroup

*
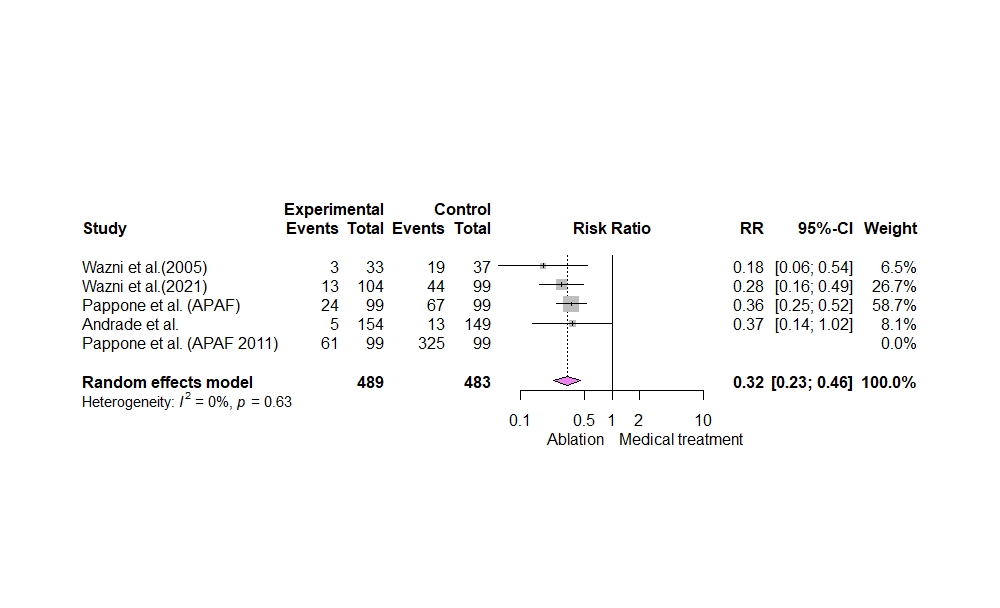
*

**Figure s15.** Forest plot of studies comparing Ablation versus medical therapy in term of hospitalization events in paroxysmal AF subgroup

*
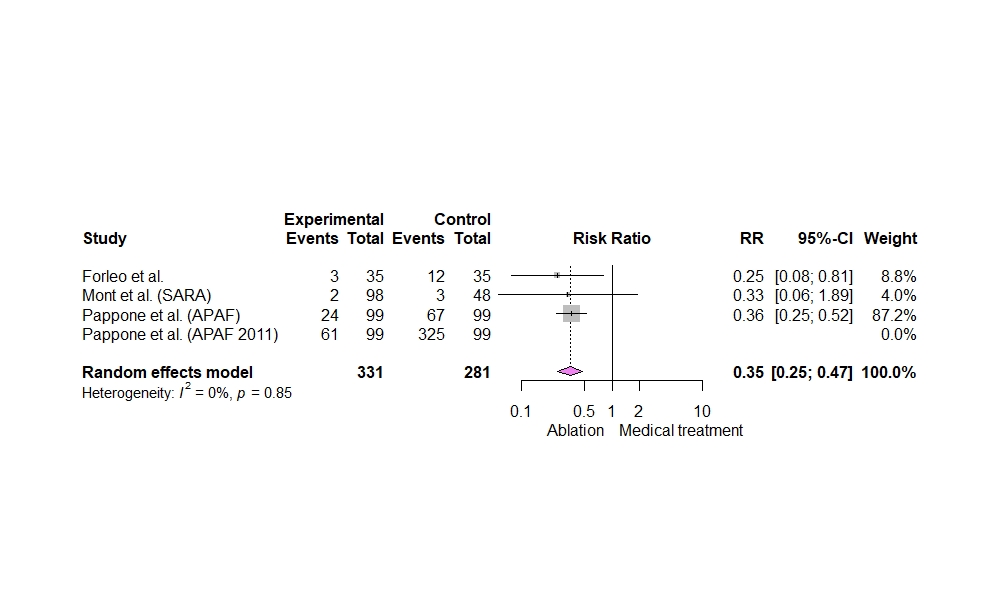
*

**Figure s16.** Forest plot of studies comparing Ablation versus medical therapy in term of hospitalization events in refractory AF subgroup

*
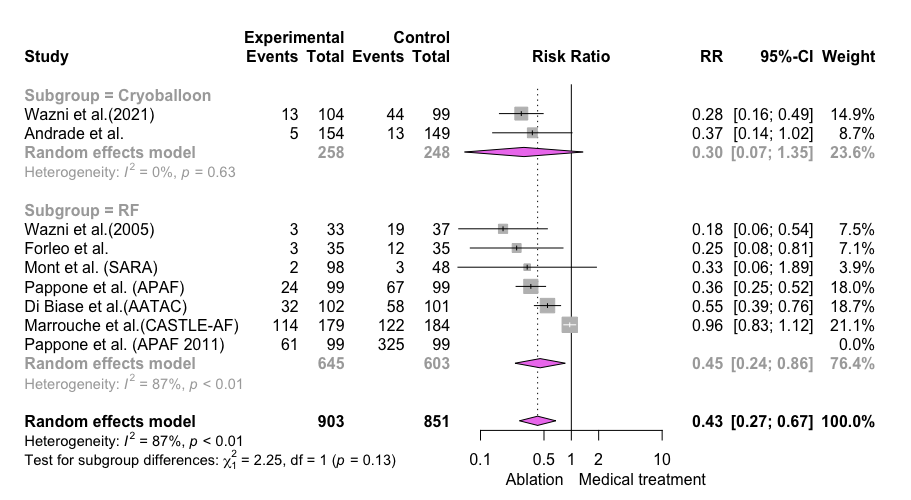
*

**Figure s17.** Forest plot of studies comparing two different methods of ablation (RF CA and Cryo-balloon CA) versus medical therapy in term of hospitalization events in AF patients

*LVEF improvement*

*
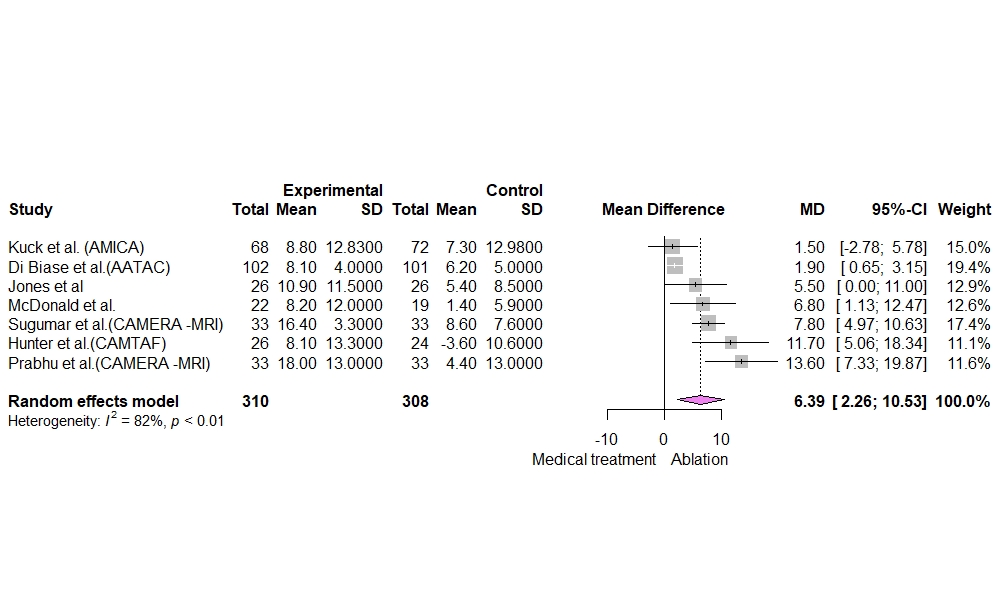
*

**Figure s18.** Forest plot of studies comparing Ablation versus medical therapy in term of LVEF improvement in persistent AF and heart failure subgroup
